# Supplementary material for: Phase II trial of cytarabine and mitoxantrone with devimistat in acute myeloid leukemia
Source: Nat Commun. 2022 Mar 30;13:1673. doi: 10.1038/s41467-022-29039-4 (PMC8967916; doi:10.1038/s41467-022-29039-4)

Supplementary Tables

Supplementary Table 1. Phase II Cohort Demographics

**Median Age (range)**	64 (21-80)
**Male**	58%
**Performance Status (range)**	1 (0-3)
**Line of Salvage (range)**	1 (1-4)
**Refractory Disease**	29%
**Therapy-related AML**	4%
**Antecedent hematologic disorder**	13%
**Prior Allogeneic Transplant**	4%
**Prior HDAC Based Salvage**	13%
**Prior HMA**	27%
**Median Duration of CR1 (months)**	11.5
**Cytogenetic Risk**	
**Poor**	38%
**Intermediate**	52%
**Good**	4%
**Unknown**	6%

Supplementary Table 2. Phase II Summary of Toxicities by Dose of Devimistat (N=48 participants)

**Dose (per m^2)**	**2,500 mg (n=7)**						
	**CTC Toxicity Grade**						
**Body System**	**1**	**2**	**3**	**4**	**5**	**Total**	**% Patients**
Blood and lymphatic system disorders	0	7	10	1	0	18	100
Cardiac disorders	7	2	0	0	0	9	85.7
Constitutional Symptoms	0	0	0	0	0	0	0
Ear and labyrinth disorders	1	0	0	0	0	1	14.3
Eye disorders	3	0	0	0	0	3	42.9
Gastrointestinal disorders	13	23	11	0	0	47	100
General disorders and administration site conditions	15	10	9	0	0	34	100
Hepatobiliary disorders	0	0	0	0	0	0	0
Immune system disorders	0	0	0	0	0	0	0
Infections and infestations	0	1	7	0	0	8	57.1
Injury, poisoning and procedural complications	1	0	0	0	0	1	14.3

Metabolism and nutrition disorders	27	15	18	1	0	61	100
Musculoskeletal and connective tissue disorders	3	12	5	0	0	20	85.7
Neoplasms benign, malignant and unspecified (incl cysts and polyps)	0	0	0	0	0	0	0
Nervous system disorders	12	4	4	0	0	20	100
Psychiatric disorders	2	10	0	0	0	12	71.4
Renal and urinary disorders	6	4	1	1	0	12	71.4
Reproductive system and breast disorders	1	0	0	0	0	1	14.3
Respiratory, thoracic and mediastinal disorders	18	0	4	0	0	22	100
Skin and subcutaneous tissue disorders	11	1	0	0	0	12	85.7
Surgical and medical procedures	0	0	0	0	0	0	0
Vascular disorders	0	7	0	0	0	7	85.7
Total	147	111	74	31	0	363	100

**Dose (per m^2)**	**2,000 mg (n=20)**						
	**CTC Toxicity Grade**						
**Body System**	**1**	**2**	**3**	**4**	**5**	**Total**	**% Patients**
Blood and lymphatic system disorders	0	19	32	1	0	52	100
Cardiac disorders	27	10	0	0	2	39	75
Constitutional Symptoms	0	1	0	0	0	1	5
Ear and labyrinth disorders	2	0	0	0	0	2	10
Eye disorders	6	1	0	0	0	7	25
Gastrointestinal disorders	62	38	22	1	0	123	100
General disorders and administration site conditions	39	26	11	0	0	76	95
Hepatobiliary disorders	0	0	0	0	0	0	0
Immune system disorders	0	0	0	0	0	0	0
Infections and infestations	1	12	23	3	0	39	85
Injury, poisoning and procedural complications	7	0	0	0	0	7	30
Metabolism and nutrition disorders	76	52	33	5	0	166	100
Musculoskeletal and connective tissue disorders	14	10	8	0	0	32	75
Neoplasms benign, malignant and unspecified (incl cysts and polyps)	1	0	0	0	0	1	5
Nervous system disorders	32	12	5	0	0	49	90
Psychiatric disorders	14	19	0	0	0	33	80
Renal and urinary disorders	15	15	2	0	0	32	60
Reproductive system and breast disorders	0	0	0	0	0	0	0

Respiratory, thoracic and mediastinal disorders	47	14	13	5	0	79	90
Skin and subcutaneous tissue disorders	39	5	1	0	0	45	90
Surgical and medical procedures	0	0	0	0	0	0	0
Vascular disorders	0	5	5	0	0	10	50
Total	446	271	166	92	2	977	100

**Dose (per m^2)**	**1,500 mg (n=21)**						
	**CTC Toxicity Grade**						
**Body System**	**1**	**2**	**3**	**4**	**5**	**Total**	**% Patients**
Blood and lymphatic system disorders	1	19	26	1	0	47	100
Cardiac disorders	8	7	5	2	1	23	57.1
Constitutional Symptoms	1	1	0	0	0	2	9.5
Ear and labyrinth disorders	4	0	1	0	0	5	23.8
Eye disorders	9	3	0	0	0	12	28.6
Gastrointestinal disorders	68	27	9	0	0	104	100
General disorders and administration site conditions	45	39	8	0	2	94	100
Hepatobiliary disorders	0	0	2	0	0	2	9.5
Immune system disorders	0	1	0	0	0	1	4.8
Infections and infestations	7	11	10	7	1	36	90.5
Injury, poisoning and procedural complications	5	1	0	0	0	6	23.8
Metabolism and nutrition disorders	67	48	30	9	0	154	100
Musculoskeletal and connective tissue disorders	18	19	7	0	0	44	76.2
Neoplasms benign, malignant and unspecified (incl cysts and polyps)	2	0	0	0	0	2	9.5
Nervous system disorders	34	19	5	0	1	59	76.2
Psychiatric disorders	14	22	5	0	0	41	76.2
Renal and urinary disorders	17	11	4	6	0	38	52.4
Reproductive system and breast disorders	2	2	0	0	0	4	14.3
Respiratory, thoracic and mediastinal disorders	33	19	8	8	0	68	81
Skin and subcutaneous tissue disorders	18	4	3	0	0	25	47.6
Surgical and medical procedures	0	1	0	0	0	1	4.8
Vascular disorders	7	8	8	0	0	23	76.2
Total	414	284	149	117	5	969	100

Supplementary Table 3 Primer Sequences Used

Gene	Sequence
Mouse Pdha:	TGCCGCTGTATCCCGCGTGT
Human PDHA:	GAAATGTGACCTTCACCGGC
Mouse ATG5:	AAGATGTGCTTCGAGATGTG
Mouse Rosa26:	GAAGATGGGCGGGAGTCTTC

Supplementary Figures:

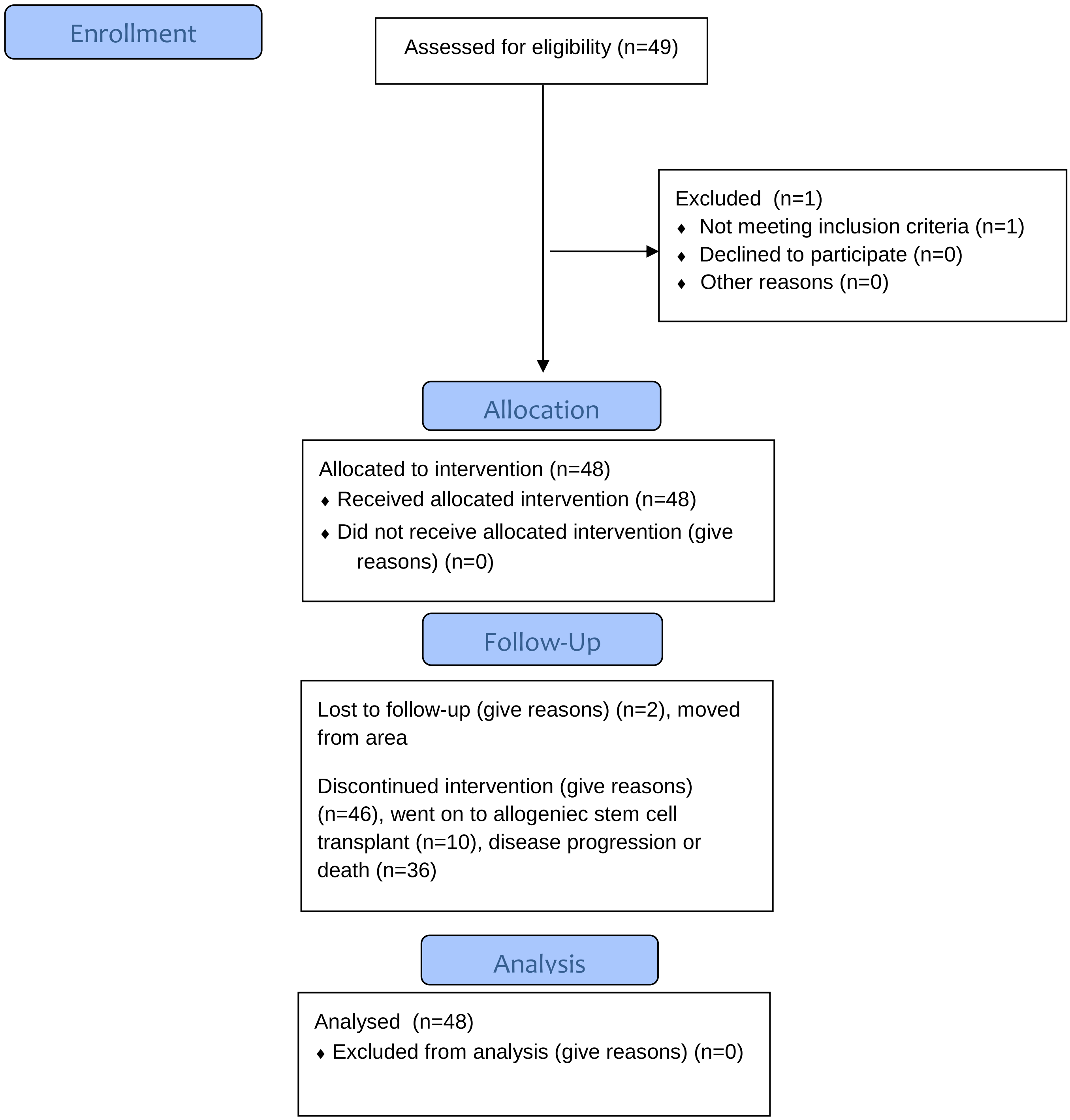

Supplementary Figure 1. Phase II CONSORT Flow Diagram.

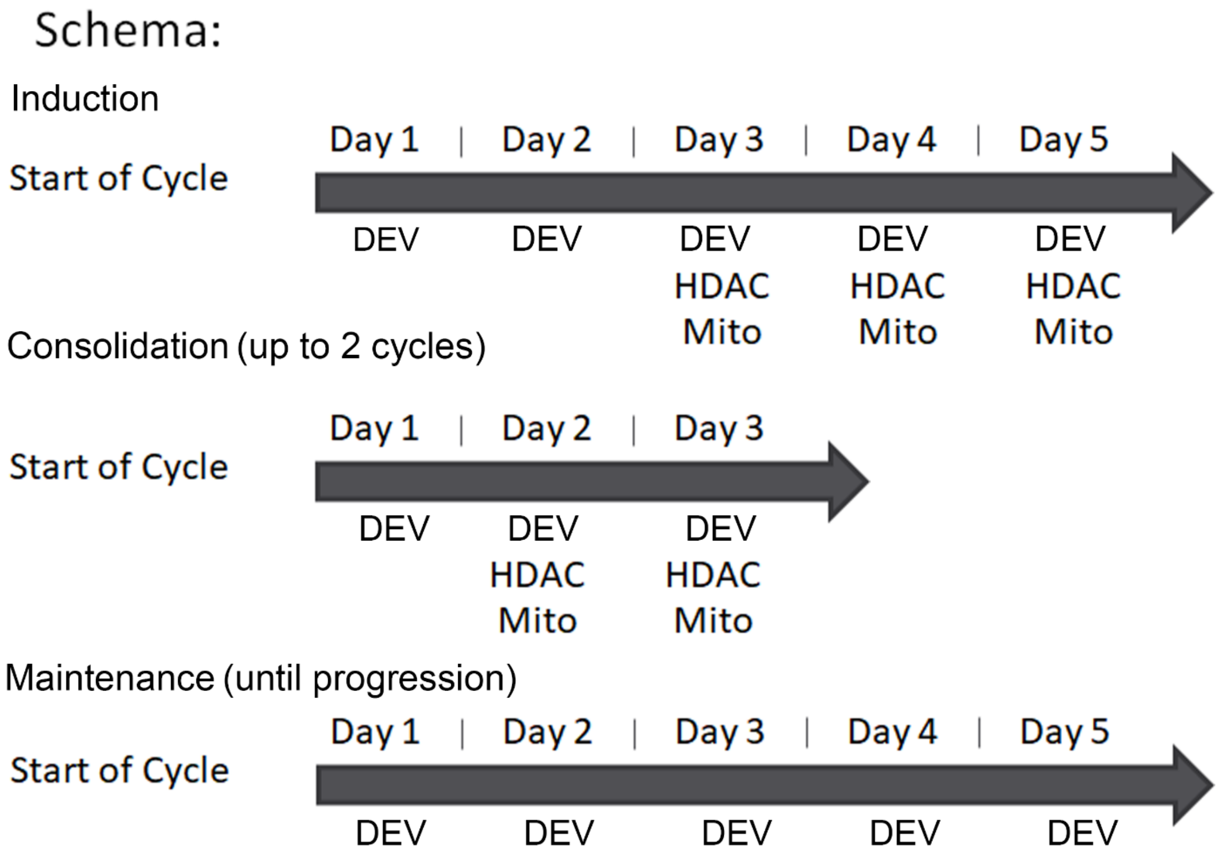

Supplementary Figure 2. Treatment schema. Induction, consolidation and maintenance therapy schedules are depicted above. Patients could receive up to 2 cycles of induction and consolidation followed by 28-day maintenance cycles until progression, transplant or withdrawal of consent. HDAC=high dose cytarabine, Mito=mitoxantrone, DEV=devimistat.

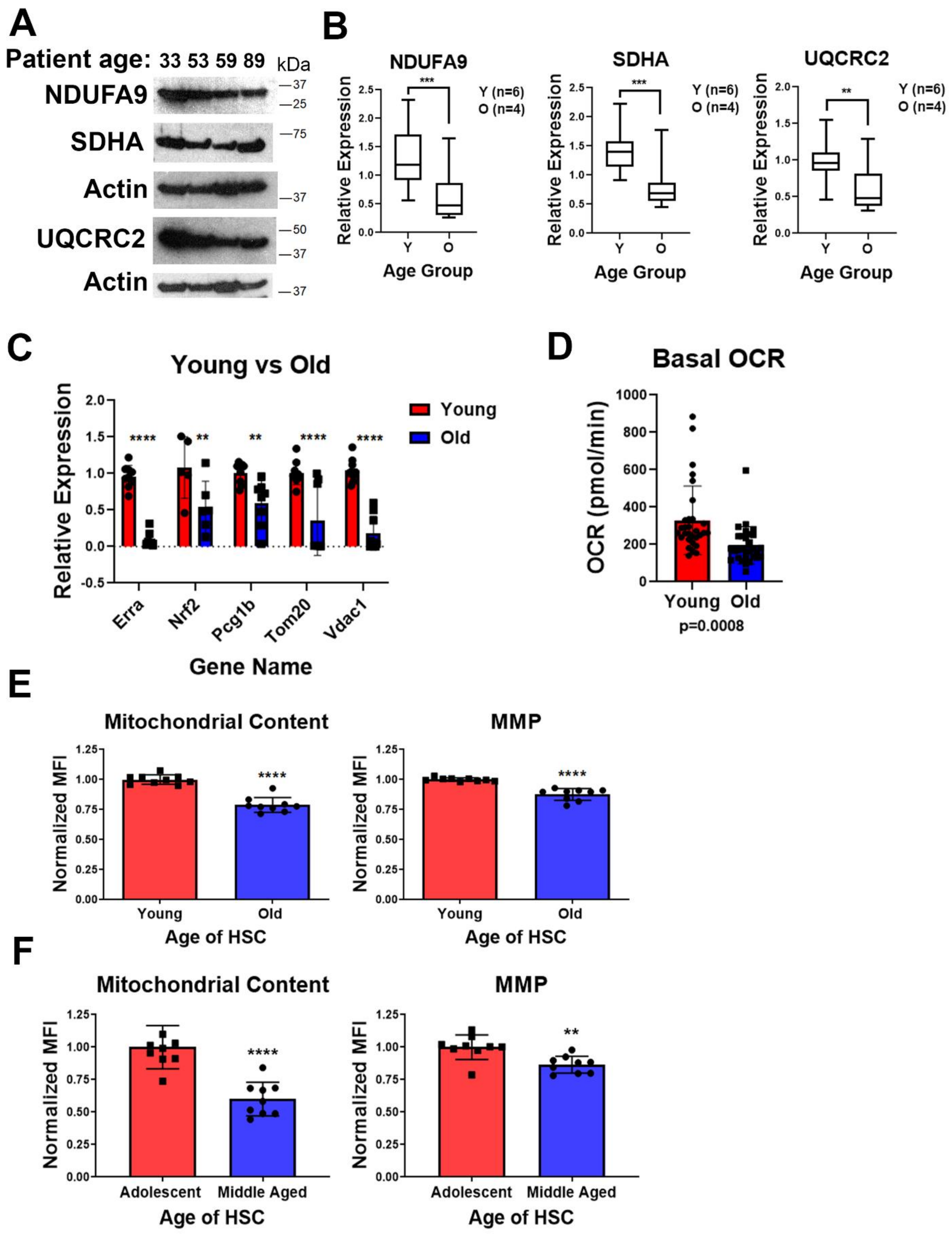

Supplementary Figure 3. AML from older HSCs have impaired mitochondrial compartments. (A) Western blots for electron transport complex members. Patient samples taken from patients of differing

ages were blotted for NDUFA9 (complex I), SDHA (complex 2) or UQCRC2 (complex 3)**.** (B) Densitometry of triplicate Western blots done as in (A) normalized to actin. Younger cohort (Y) consisted of 6 patients younger than 50 years of age and older cohort (O) consisted of 4 patients older than 50. Whiskers represent min to max values, mean is indicated by the horizontal line and box height represents the interquartile distance. (C) Gene expression of mitochondrial related genes from AML cells derived from young or old murine HSCs. Shown are the means of three biological replicates each attempted in triplicate. Means were compared by two sided ANOVA with a Sidak's posttest for multiple comparisons. (D) Basal oxygen consumption rates of AML cells derived from young or old murine HSCs. Shown are the means of three biological replicates each done with 10 replicates. (E) Mitochondrial staining of AML cells derived from young or old murine HSCs. TMRM (MMP) and mitotracker (mitochondrial content) staining of AML cells derived from young or old murine HSCs. Shown are the means of three biological replicates each done in triplicate. (F) Mitochondrial staining of AML cells derived from adolescent (6 weeks old) or middle aged (15 months old) murine HSCs. TMRM and mitotracker staining as in (E). All mitochondrial staining studies were done in triplicate in at least 3 independent experiments. Where two means are compared p value calculated by students 2 tailed t test. When more than two means were compared an ANOVA analysis with Sidak's multiple comparisons correction was done. *=p<0.05, **=p<0.01, ***=p<0.005 ****=p<0.001. All error bars represent the standard deviation around the mean.

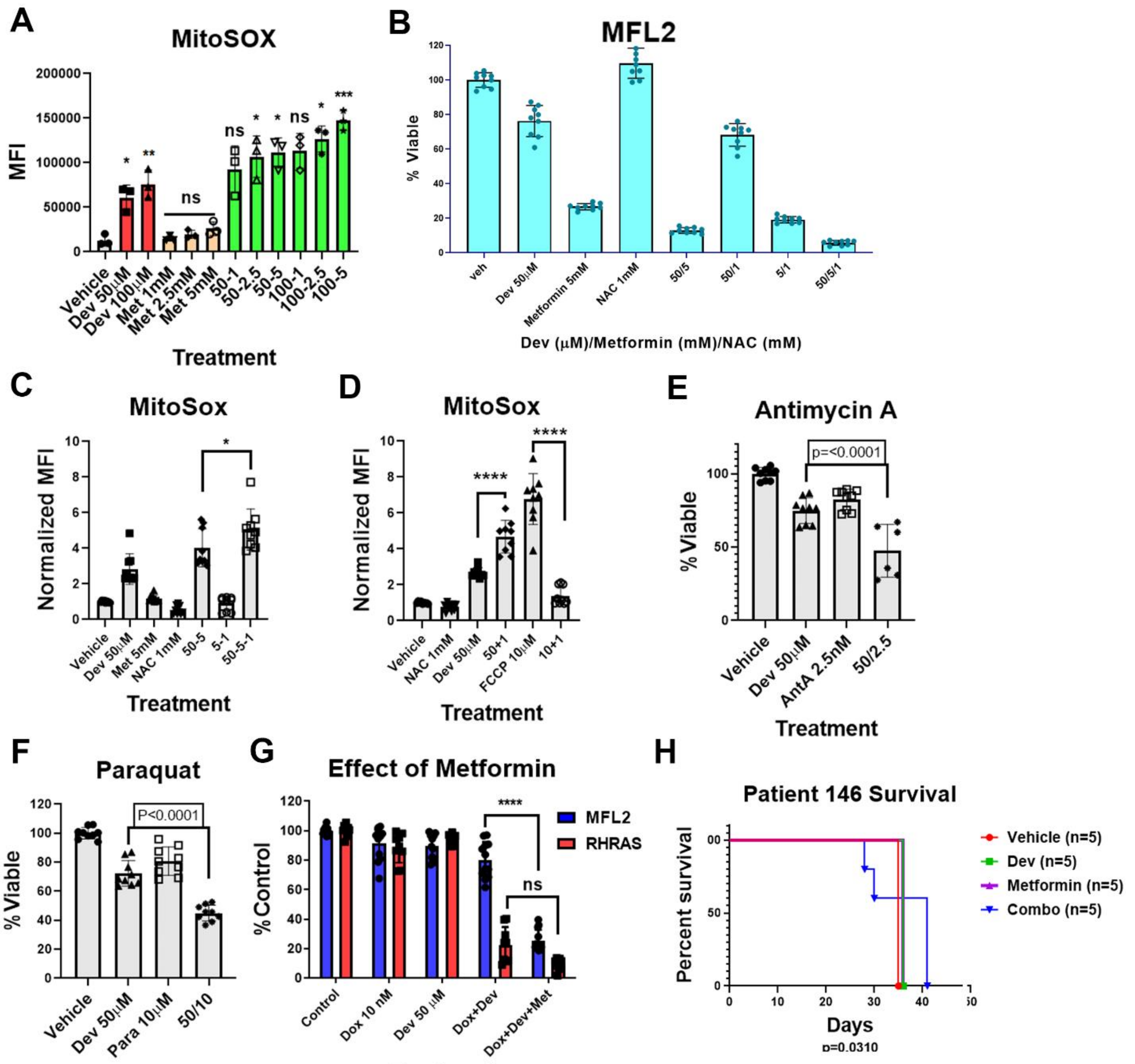

Supplementary Figure 4. Effects of MMP and Reactive Oxygen Species on Devimistat. (A) Mitochondrial ROS generated by devimistat or metformin or both. MFL2 cells were treated as indicated for 24 hours and stained for mitochondrial ROS. (B) Viability assays. MFL2 cells were treated as indicated for 72 hours and viability assessed. Dev=devimistat, NAC=n-acteyl-cysteine. (C) Mitochondrial ROS generated by devimistat, metformin and NAC. MFL2 cells were treated as indicated for 24 hours and stained for mitochondrial ROS. (D) Mitochondrial ROS generated by devimistat, FCCP with and without NAC. MFL2 cells were treated as indicated for 24 hours and stained for mitochondrial ROS. (E+F) (D+E) Viability assays. MFL2 cells were treated with the indicated amounts of devimistat (Dev), antimycin A (AntA), paraquat (Para) or the combination for 72 hours and viability assessed. (G) Viability assays. MFL2 and RHRAS cells were treated with the indicated amounts of doxorubicin (Dox), devimistat (Dev) with and without 5mM metformin (Met) for 72 hours and viability assessed. (H) PDX

model derived from a 75 year old AML patient was injected into NSG mice which were then treated with vehicle, devimistat, metformin or the combination and followed for survival. For all viability assays a mean of three independent experiments each done in triplicate are shown. . *=p<0.05, **=p<0.01, ***=p<0.005, ****=p<0.001, ns=p>0.05. All error bars represent the standard deviation around the mean.

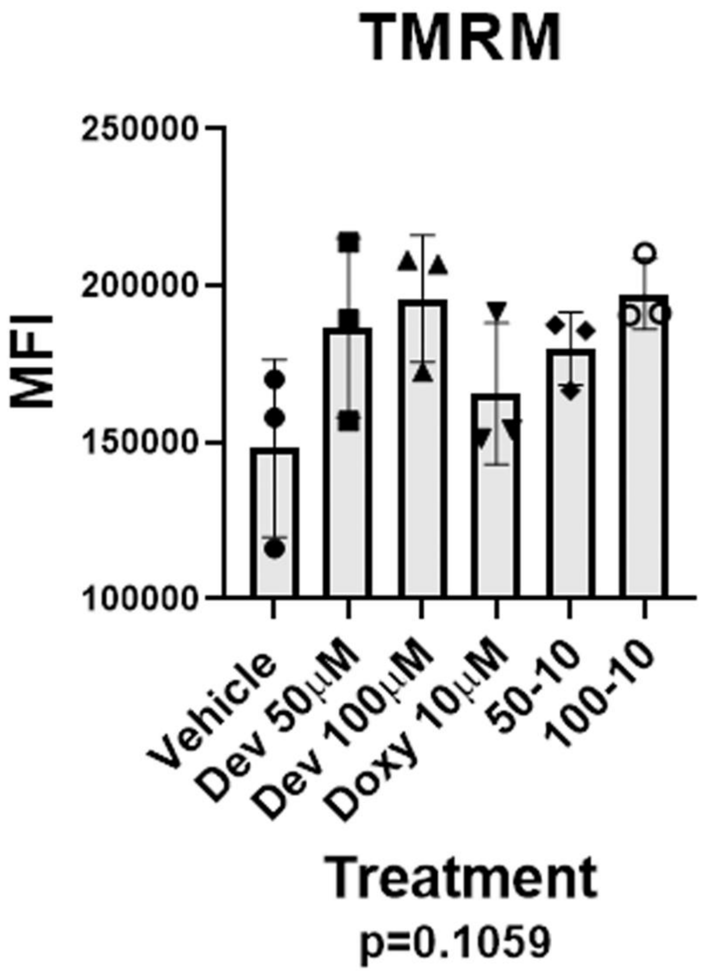

Supplementary Figure 5. Doxycycline does not effect MMP. MFL2 cells were treated as indicated for 24 hours and MMP determined by TMRM staining. Shown are means from 3 independent experiments. P value was calculated by a two tailed ANOVA analysis. All error bars represent the standard deviation around the mean.

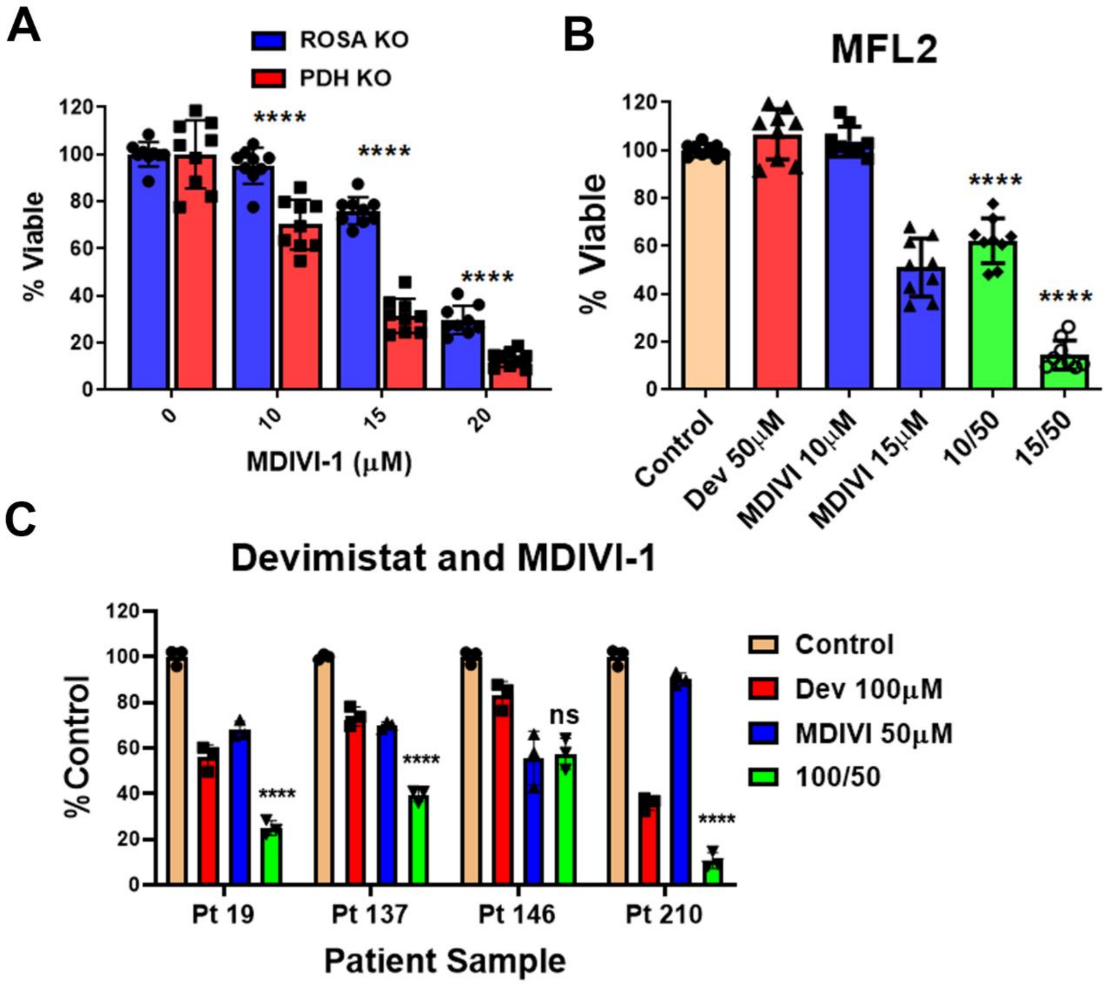

Supplementary Figure 6. AML cells are dependent on Drp1 during TCA cycle inhibition. (A) Viability assay. Pdha1 deleted (PDH KO) or control (ROSA KO) cells were incubated with the indicated concentration of MDIVI-1 for 72 hours and assessed for viability. (B) Viability assay. MFL2 cells were incubated with devimistat (CPI), MDIVI-1 (MDIVI) or both at the indicated μM concentrations for 72 hours and viability assessed. (C) Primary patient sample derived AML cells were placed in culture and treated as indicated for 72 hours. Following treatment cells were stained with annexinV and PI and analyzed by flow cytometry. Each treatment was done in triplicate. For all viability assays a mean of three independent experiments each done in triplicate are shown. Means compared by 2 way ANOVA and multiple comparisons corrected by Sidak's posttest. ****=p<0.001. All error bars represent the standard deviation around the mean.

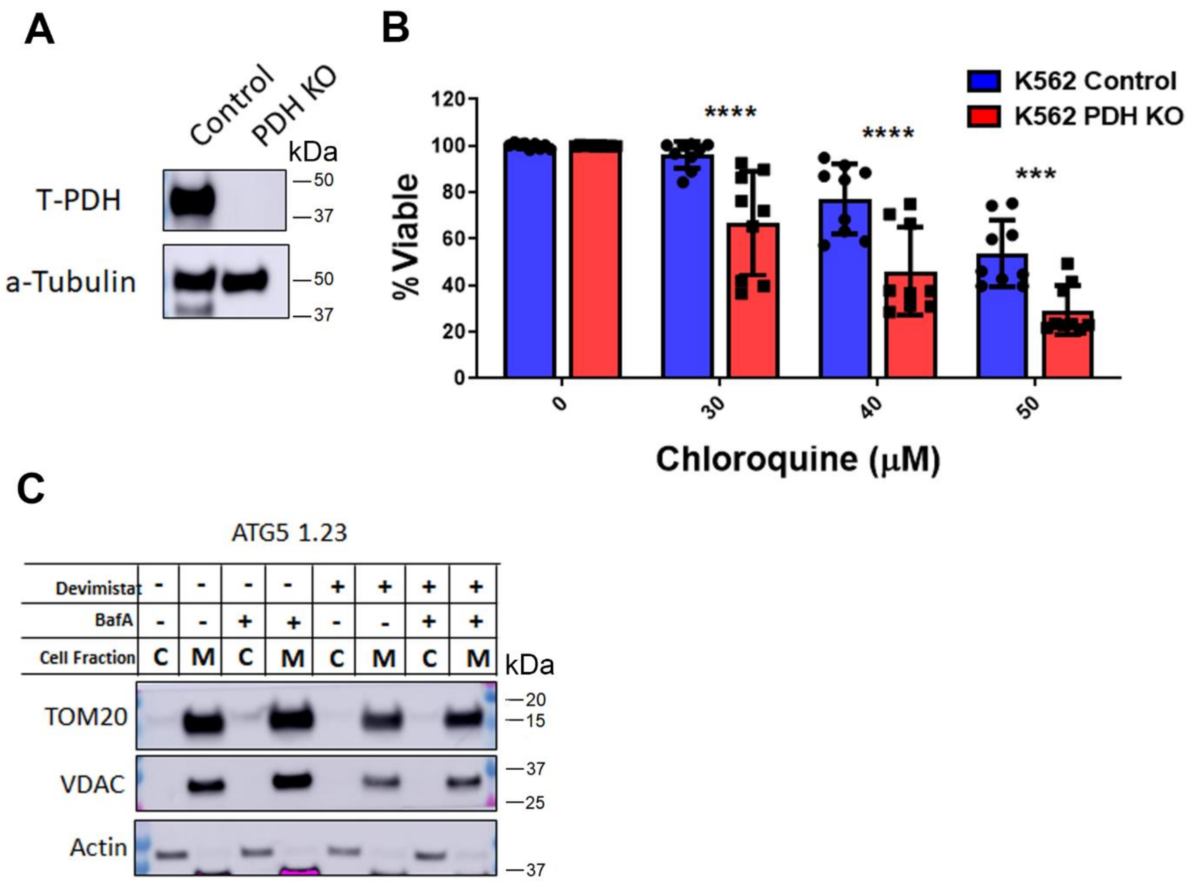

Supplementary Figure 7. Autophagy inhibition with chloroquine or deletion of ATG5 sensitize cells to devimistat. (A) Western blot. K562 cells expressing Cas9 were infected with sgRNAs targeting the E1α subunit of PDH (*PDHA1*) or a non-targeting control. Clonal isolates were obtained by serial dilution and blotted for PDHA1. Tubulin is used as a loading control. (B) Viability assays. Clones from figure S1A were incubated in the indicated concentrations of chloroquine for 72 hours and viability assessed. (C) Western blot for Vdac1 and Tom20. *Atg5* KO cells were treated with 50 µM devimistat for 24 hours, lysed and cytosolic and mitochondrial fractions blotted for the indicated protein. Actin was used as a localization and loading control. For viability experiments a mean of three independent experiments each done in triplicate are shown. Means compared by 2 way ANOVA and multiple comparisons corrected by Sidak's post test. ****=p value of 0.001 or less. All error bars represent the standard deviation around the mean.

Flow Cytometry Viable Cell Gating Strategy example:

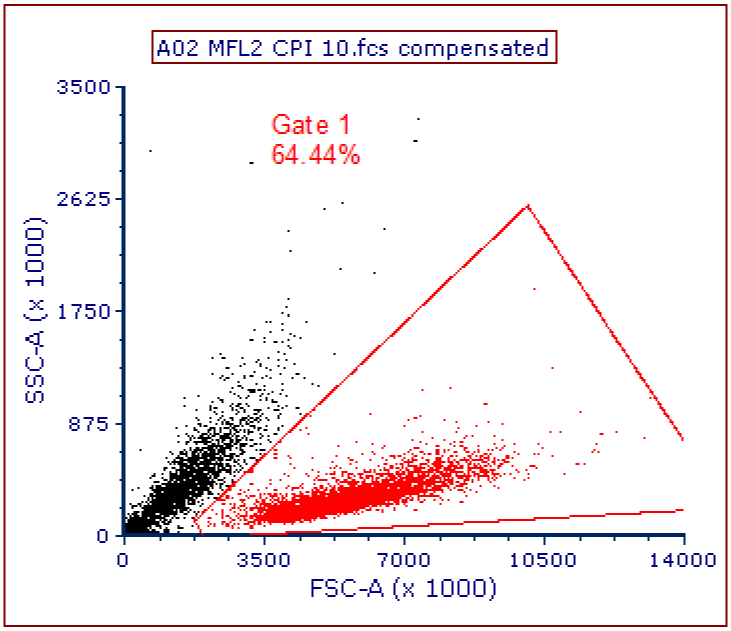

Supplementary Note 1.

Complete Phase II Protocol

1.0 Introduction and Background

1.1 Proposed Mechanism of Action

CPI-613, the investigational drug, is a novel anti-tumor compound presumably with a novel mechanism of action that does not belong to any existing pharmacological class of anticancer agents currently used in the clinics. Specifically, CPI-613 is referred to as an Altered Energy Metabolism-Directed (AEMD) compound, and it is selective against tumor cells (but not normal cells) according to preclinical studies. The anti-tumor effects of CPI-613 are believed to be exerted by the inhibition of the pyruvate dehydrogenase complex (PDC).

In both normal and tumor cells, energy is derived from glucose, first by breaking down glucose to pyruvate via the glycolytic cycle in the cell cytoplasm. Once formed in mammalian cells, pyruvate is then converted by PDC to acetyl-CoA that is in turn metabolized via the tricarboxylic acid (TCA) cycle in the mitochondria.

Most tumor cells display profound perturbation of energy metabolism that tightly correlates with malignant transformation (Baggetto 1992). The PDC is regulated by the phosphorylation state of the E1 subunit. When phosphorylated by the PDC kinase the enzyme is inhibited and the conversion of pyruvate to acetyl-CoA is inhibited. Preclinical studies have shown that CPI-613 causes phosphorylation of the E1 subunit in tumor cells with little to no effect on normal cells. As a result of this phosphorylation ATP levels in tumors cells fall and cancer cell death ensues.

Consistent with the proposed mechanism is the fact that CPI-613 has been shown to have anti-tumor activity in cell culture and animal tumor models against diverse cancers independent of multiple drug resistance, cell cycle phase and activated signal transduction pathways. The novelty in the presumed mechanism of action for CPI-613 is further supported by the results from an *ex vivo* study demonstrating that CPI-613 is effective against various types of tumor cells excised from patients that were resistant to different anti-cancer drugs currently used in the clinics.

The significance of CPI-613 having a novel mechanism of action that is not shared by any existing pharmacological class of anti-cancer agents currently used in the clinics is that CPI-613 may be effective not only against naive tumors that have never been treated with any anti-cancer agents, but also effective against tumors that are resistant to anti-cancer agents currently used in the clinics. This is an important aspect because tumors frequently develop resistance to anti-cancer agents, rendering a shortage of treatment options. The availability of a novel anti-cancer agent would provide a new tool to treat cancer.

1.2 Background on Preclinical Studies

CPI-613 is being developed as an anti-tumor agent for the treatment of cancer. A number of preclinical anti-tumor efficacy studies; Administration, Distribution, Metabolism, and Elimination (ADME) studies; safety pharmacology studies; and toxicity studies of CPI-613 have been conducted. These studies are briefly described below, and the Investigator's Brochure provides detailed information regarding these preclinical studies.

In Vitro and Animal Efficacy Studies

In vitro cell culture studies have shown that CPI-613 is effective against a variety of tumor cell lines such as human BxPC-3 pancreatic tumors, human AsPC-1 pancreatic tumors, human H-460 non-small cell lung carcinoma (NSCLC), and human A2780 ovarian tumors, as well as explants of different types of fresh human tumors including NSCLC, colon, breast, and pancreatic tumors. Additionally the drug has activity against human AML cell lines including MV4-11 and HL60. Additional work has shown that the immortalized NIH 3T3 cell line is not affected by CPI-613. Once these cells have been transformed by oncogeneic RAS expression they become sensitive. This is a mutation commonly found in hematologic malignancies including the acute leukemias. Consistent with the *in vitro* studies are animal efficacy

studies, which showed that CPI-613 was effective against xenograft of human BxPC-3 and AsPC-1 pancreatic tumors, and human H-460 NSCLC.

Additionally, *in vitro* cell culture studies have shown that CPI-613 was equally effective against tumor cell lines with well-characterized mechanisms of drug resistance and their parental tumor cell lines. The equal effectiveness against resistant and parental tumor cell lines is consistent with the proposed novel mechanism of action of CPI-613 that is not shared by any chemotherapeutic agents currently used in the clinics. The drug-resistant tumor cell lines and the parental studied are listed below:

**Parental Tumor Cell Lines**	**Drug-Resistant Derivative**
MCF-7 breast carcinoma line	NCI/ADR-RES adriamycin-resistant derivative(Recently reclassified as an Ovarian Cancer derivative of OVCAR-8 FYI)
A2780 ovarian carcinoma cell line	A2780DX doxorubicin-resistant derivative
NCI/H69 small cell lung cancer cells	H69-AR anthracycline-resistant derivative
MES-SA uterine sarcoma cells	MES-SA/MX2 mitoxanthrone-resistant derivative

Pharmacokinetic Studies

In rats, the toxicokinetic profile of CPI-613 administered 2x weekly for 3 weeks by IV administration at dose levels of 25, 30 or 35 mg/kg (i.e., 150, 180 or 210 mg/m^2, respectively) was characterized by an apparent biphasic profile – an initial distribution phase followed by an elimination phase. Systemic exposure was proportional to dose level, and there was no evidence of test article accumulation or sex differences in exposure. In minipigs, the toxicokinetic profile of CPI-613 administered 2x weekly for 3 weeks by IV administration at dose levels of 45, 42 and 46 mg/kg (i.e., 1575, 1470 and 1617 mg/m^2, respectively) was also characterized by an apparent biphasic profile.There was also no evidence of test article accumulation or sex differences in exposure.

ADME Studies

A PK study in minipigs showed that the log scale of the half-life (t1/2) values tended to be directly and linearly related to the log of the dose. Specifically, t1/2 was ~1hour at 3 mg/kg (105 mg/m^2), ~1.7 hours at 10 mg/kg (350 mg/m^2), and 10 hours at 30 mg/kg (1050 mg/m^2).

The tissue distribution pattern of radioactivity, after an intravenous (IV) administration of ^{14}C-labeled CPI-613 in rats showed that:

1. The liver might be involved in the metabolism of CPI-613 and/or ^{14}C-containing metabolites of CPI-613.
2. Both the GI tract and the kidneys might be involved in the elimination of CPI-613 and/or ^{14}C-containing metabolites of CPI-613.
3. CPI-613 and 14C-containing metabolites of CPI-613 were mostly present in the extracellular space in the circulation.

An *in vitro* human plasma protein binding study showed that CPI-613 exhibited 99.7% binding to human plasma proteins, a value that was similar to warfarin. These results are consistent with findings from tissue distribution studies (see above) which showed CPI-613 was mostly present in the extracellular space in the circulation, consistent with findings of extensive plasma protein binding.

An *in vitro* human CYP450 enzyme inhibition study showed CPI-613 does not significantly inhibit various CYP450 isozymes, including 1A2, 2C9, 2C19, 2D6, and 3A4.

An *in vitro* human liver S9 fraction hepatocyte study showed that CPI-613 was oxidized to form a sulfoxide metabolite rapidly, with a half-life of <19 minutes. CPI-613 was also glucuronidated rapidly at the terminal carboxyl group, with a half-life of <15 minutes. Due to oxidation and glucuronidation, potential metabolites are a sulfoxide, a metabolite with glucuronidation in the terminal carboxyl group; and/or a metabolite with combination of sulfoxidation and glucuronidation.

In Vitro and Animal Pharmacology Safety Studies

An *in vitro* study showed that CPI-613 did not inhibit Ether-a-Go-Go-Related Gene (hERG) channels. Since hERG channel inhibition is the predominant cause of QTc interval prolongation which has been linked to Torsades de Pointes conditions such as sudden cardiac death and ventricular arrhythmia, this study suggested that CPI-613 may not induce Torsades de Pointes conditions. This is consistent with the subsequent animal toxicology studies showing that CPI-613 did not induce abnormal ECG pattern.

The potential influence of air in the dead space of an IV catheter on the acute toxicity of CPI-613 in rats has been evaluated. The results suggested that standard drug administration procedures in the elimination of dead air space within indwelling IV catheters should be followed, since air in the catheter air space may enhance the acute toxicity of CPI-613 at supra-antitumor doses.

The acute toxicity of CPI-613 administered IV as a bolus vs. by infusion has been compared in a preliminary study in rats. The results suggested that CPI-613 at supra-antitumor doses might be less toxic when administered as a 1-hr IV infusion than as a 1-min IV bolus.

Animal Toxicology Studies

Single Dose: A toxicology study involving a single IV administration of CPI-613 in mice has been conducted. The results indicated that 0.3-3 mg/kg (0.9-9 mg/m²) of CPI-613 did not induce any toxicity, whereas 10-100 mg/kg (30-300 mg/m²) also did not induce toxicity, except inflammatory reactions at or around the site of injection with intensity that was dose-related.

Twice Weekly for 1 Week Dosing: Animal toxicology studies involving IV administrations of CPI-613 twice weekly for 1 week have been conducted in mice and minipigs. In mice, CPI-613 at 100 mg/kg (300 mg/m²) induced mortality in all female mice but not male mice. Also, CPI-613 at 30 and 75 mg/kg (90 and 215 mg/m², respectively) did not induce any toxicity, except inflammation around the site of injection and a small increase in the spleen weight in mice treated with 75 mg/kg (215 mg/m²) and in the surviving mice treated with 100 mg/kg (300 mg/m²) of CPI-613.

In minipigs, when given 2x weekly for 1 week, CPI-613 at doses up to 30 mg/kg (1,050 mg/m²), the highest dose investigated in this study, did not induce toxicity, other than local inflammatory reactions at and around the site of injection. These results are consistent with those observed with the mouse study (see above).

Twice Weekly for 3 Weeks Dosing: Animal toxicology studies involving IV administrations of CPI-613 twice weekly for 3 weeks have been conducted in rats and minipigs, as described below.

In rats, when administered at dose levels of 25, 30 or 35 mg/kg (i.e., 150, 180 or 210 mg/m², respectively), CPI-613 enhanced inflammatory responses in the body in a dose related manner, as reflected by dose-related exaggeration in the incidence and severity of granulomatous or acute, chronic, or chronic active inflammation at the administration site inguinal vein), skin and subcutaneous tissue near the port, and inguinal skin and subcutaneous tissue near the inguinal vein, when compared to control treatment. CPI-613 at the toxicological doses of 30-35 mg/kg (180-210 mg/m²) in rats induced dose-related incidence of mortality. The cause of death was due to significant systemic inflammation, and might be related to an over enhancement of the inflammatory responses induced by CPI-613. Deaths due to significant systemic inflammation were also observed in minipigs treated with the minipig toxicological dose of 46.2 mg/kg (i.e., 1617 mg/m²) of CPI-613 in another toxicology study. CPI-613 at 25, 30 and 35 mg/kg (i.e., 150, 180 and 210 mg/m², respectively) elevated GGT levels in a dose-dependent manner in rats. This elevation is not associated with histopathology findings or other parameters of kidney or liver functions, and therefore not related to toxicity of the kidney or liver. However, it may be related to systemic inflammation (Yamada J, Tomiyama H, *et. al.* 2006), since systemic inflammation was observed in CPI-613 treated rats in this study. Finally, CPI-613 at the rat toxicological doses of 30 and 35 mg/kg (i.e., 180 and 210 mg/m², respectively) increased reticulocyte levels, which appeared to be associated with hematopoietic cell proliferation of the spleen, and sternal bone marrow hyperplasia that were secondary to inflammation initiated at the administration site or port in rats. The no-observable-adverse effect levels were 25 mg/kg (150 mg/m²) CPI-613. The LD10 or STD10 is approximately 35 mg/kg (210 mg/m²).

In minipigs, when administered at dose levels of 42, 45, 46.2, 50 and 55 mg/kg (i.e., 1470, 1575, 1617, 1750 and 1925 mg/m², respectively), CPI-613 at all dose levels induced transient vomiting that was transient and reversible. Mortality (2 of 8 minipigs) was observed in minipigs treated with CPI-613 at 46.2 mg/kg (i.e., 1617 mg/m²), due to significant systemic inflammation. Significant systemic inflammation was also observed in rats treated with CPI-613, resulting in dose-dependent incidence of mortality at the toxicological doses of 30-35 mg/kg (180-210 mg/m²) and might be due to an over enhancement of inflammatory responses induced by toxic doses of CPI-613, as supported by dose-related exaggeration in inflammatory effects according to gross necropsy and histopathology findings (see below).

Overall Summary of Preclinical Studies

Preclinical efficacy studies, ADME studies, tissue distribution studies, and toxicology studies on CPI-613 have been conducted. The efficacy studies demonstrated the anti-tumor effects of CPI-613, whereas the ADME studies provided PK, biodistribution, metabolism and plasma protein binding

information on CPI-613 in the body. Finally, for the toxicology studies, CPI-613 was administered in a way that mimicked that in the proposed clinical study, which involved IV administration given twice weekly. The results of the animal toxicology showed that CPI-613 is sufficiently non-toxic.

1.3 Background on Clinical Studies

Six single-patient physician-sponsored clinical studies have been conducted for compassionate use. Three of these single-patient studies were conducted in the US, whereas the other 3 were conducted in Israel.

Additionally, a Phase I clinical study involving CPI-613 as a single agent in patients with solid tumors and lymphomas is ongoing. A phase I clinical study involving CPI-613 as a single agent in patients with advanced hematologic malignancies is ongoing. A phase I study of CPI-613 in combination with high dose cytarabine and mitoxantrone has recently been completed. This trial established the MTD of CPI-613 as 2,500 mg/m^2. A Phase I/II clinical study involving CPI-613 in combination with Gemcitabine is ongoing.

1.4 Rationale

Chemotherapy resistance is a major cause of death in patients with relapsed or refractory AML. CPI-613 can increase the sensitivity of leukemia cells to chemotherapy in petri dishes and mice. As a single agent in a phase I clinical trial several relapsed/refractory AML patients had an objective response. Additionally, a phase I study of CPI-613 in combination with high dose cytarabine and mitoxantrone has recently been completed. This trial established the MTD ofCPI-613 as 2,500 mg/m^2. Preliminary analysis of the study revealed an overall complete remission or complete remission with incomplete count recovery rate of 45%. This compares favorably with our historical experience of this regimen without CPI-613. In this trial patients with poor risk cytogenetics had a 46% response rate, far superior to the 19% response seen with historical data. This trial established that CPI-613 can be safely administered at 2,500 mg/m^2 in this combination and results in superior response rates. The novel mechanism of action, non-cross resistance with chemotherapeutic agents, lack of CPI-613-related myelosuppression clinically and increase response rate make this regimen a suitable candidate for the proposed pilot study.

1.5 Justification of the Dose of CPI-613

WFUCCC 22112, a phase I study of CPI-613 in combination with high dose cytarabine and mitoxantrone given exactly as proposed in the current study established the MTD of CPI-613 to be 2,500 mg/m^2. A detailed analysis of the response data indicated that similar response rates were seen at the 2,000 mg/m^2 dose with less toxicity. Further studies have indicated a dose of 1500 mg/m^2 has a similar efficacy profile as 2,000 mg/m^2 but with even less toxicity. The rationale behind using a dose of 500mg/m^2 is supported by three lines of evidence: 1) pre-clinical evidence in mice suggesting lower doses may be more efficacious overtime than higher doses, 2) a traditional dose response effect of CPI-613 is not observed in AML patients thus far, and 3) a previous study in pancreatic cancer

that showed remarkable synergy between CPI-613 at 500mg/m^2 in combination with standard chemotherapy.

2.0 Objectives

2.1 Primary Objective

To determine the feasibility of CPI-613 when administered with high dose cytarabine, and mitoxantrone in all three phases of salvage therapy (induction, and maintenance). The regimen will be considered feasible if ≥50% of patients eligible for maintenance therapy complete at least 3 cycles.

2.2 Secondary Objectives

To observe the response rate (CR, and CRi) of CPI-613 in combination with high dose cytarabine and mitoxantrone.

To observe the overall survival of patients treated with CPI-613 in combination with high dose cytarabine and mitoxantrone in induction, consolidation and maintenance.

To monitor toxicities experienced by patients treated with CPI-613 in combination with high dose cytarabine and mitoxantrone in induction, consolidation and maintenance.

3.0 Patient Selection

This clinical trial can fulfill its objective only if patients appropriate for this trial are enrolled. All relevant medical and other considerations should be taken into account when deciding whether this protocol is appropriate for a particular patient. Physicians should consider the risks and benefits of any therapy, and therefore only enroll patients for whom this treatment is appropriate.

3.1 Inclusion Criteria

3.1.1 Patients must have histologically or cytologically documented relapsed and/or refractory Acute Myeloid Leukemia or granulocytic sarcoma.

3.1.2 ECOG Performance Status of ≤3.

3.1.3 Must be ≥18 years of age.

3.1.4 Expected survival >3 months.

3.1.5 Women of child-bearing potential (i.e., women who are pre-menopausal or not surgically sterile) must use accepted contraceptive methods (abstinence, intrauterine device [IUD], oral contraceptive or double barrier device), and must have a negative serum or urine pregnancy test within 1 week prior to treatment initiation. (**Note:** Pregnant patients are excluded because the effects of CPI-613 on a fetus are unknown.)

3.1.6 Fertile men must practice effective contraceptive methods during the study period, unless documentation of infertility exists.

3.1.7 Mentally competent, ability to understand and willingness to sign the informed consent form.

3.1.8 No radiotherapy, treatment with cytotoxic agents (except CPI-613), treatment with biologic agents or any anti-cancer therapy within the 2 weeks prior to treatment with CPI-613. Hydroxyurea and oral tyrosine kinase inhibitors being used without Grade ≤ 2 toxicity can be taken until day 1 of therapy. Patients must have fully recovered from the acute, non-hematological, non-infectious toxicities of any prior treatment with cytotoxic drugs, radiotherapy or other anti-cancer modalities (returned to baseline status as noted before most recent treatment). Patients with persisting, non-hematologic, non-infectious toxicities from prior treatment ≤ Grade 2 are eligible, but must be documented as such.

3.1.9 Laboratory values ≤2 weeks must be:

- Adequate hepatic function (aspartate aminotransferase [AST/SGOT] ≤3x upper normal limit [UNL], alanine aminotransferase [ALT/SGPT] ≤3x UNL (≤5x ULN if liver metastases present), bilirubin ≤1.5x UNL).
- Adequate renal function (serum creatinine ≤1.5 mg/dL or 133 μmol/L).

3.1.10 Left Ventricular Ejection Fraction (by TTE, MUGA or cardiac MRI) sufficient to safely administer mitoxantrone as determined by the treating physician

3.2 Exclusion Criteria

3.2.1 Serious medical illness, such as significant cardiac disease (e.g. symptomatic congestive heart failure, unstable angina pectoris, myocardial infarction within the past 6 months, uncontrolled cardiac arrhythmia, pericardial disease or New York Heart Association Class III or IV), or severe debilitating pulmonary disease, that would potentially increase patients' risk for toxicity.

3.2.2 Patients with active central nervous system (CNS) or epidural tumor.

3.2.3 Albumin <2.0 g/dL or <20 g/L.

3.2.4 Any active uncontrolled bleeding, and any patients with a bleeding diathesis (e.g., active peptic ulcer disease).

3.2.5 Pregnant women, or women of child-bearing potential not using reliable means of contraception (because the teratogenic potential of CPI-613 is unknown).

3.2.6 Lactating females because the potential of excretion of CPI-613 into breast milk. (**Note:** Lactating females are excluded because the effects of CPI-613 on a nursing child are unknown.)

3.2.7 Fertile men unwilling to practice contraceptive methods during the study period.

3.2.8 Life expectancy less than 3 months.

3.2.9 Any condition or abnormality which may, in the opinion of the investigator, compromise the safety of patients.

3.2.10 Unwilling or unable to follow protocol requirements.

3.2.11 Patients with large and recurrent pleural or peritoneal effusions requiring frequent drainage (e.g. weekly). Patients with any amount of clinically significant pericardial effusion.

3.2.12 Active heart disease including symptomatic coronary artery disease, uncontrolled arrhythmias, or symptomatic congestive heart failure.

3.2.13 Evidence of ongoing, uncontrolled infection.

3.2.14 Patients with known HIV infection. (**Note:** Patients with known HIV infection are excluded because patients with an immune deficiency are at increased risk of lethal infections when treated with marrow-suppressive therapy, and because there may be unknown or dangerous drug interactions between CPI-613 and anti-retroviral agents used to treat HIV infections.)

3.2.15 Patients receiving any other standard or investigational treatment for their cancer, or any other investigational agent for any indication within the past 2 weeks prior to initiation of CPI-613 treatment (the use of Hydrea is allowed).

3.2.16 Patients who have received immunotherapy of any type within the past 4 weeks prior to initiation of CPI-613 treatment.

3.2.17 Requirement for immediate palliative treatment of any kind including surgery.

3.2.18 Patients that have received a chemotherapy regimen with stem cell support in the previous 6 months.

3.3 Inclusion of Women and Minorities

Both men and women and members of all races and ethnic groups are eligible for this trial.

4.0 Registration Procedures

All patients entered on any CCCWFU trial, whether treatment, companion, or cancer control trial, **must** be linked to the study in EPIC within 24 hours of Informed Consent. Patients **must** be registered prior to the initiation of treatment.

You must perform the following steps in order to ensure prompt registration of your patient:

1. Complete the Eligibility Checklist (Appendix A)
2. Complete the Protocol Registration Form (Appendix B)
3. Alert the Cancer Center registrar by phone, *and then* send the signed Informed Consent Form, Eligibility Checklist and Protocol Registration Form to the registrar, either by fax or e-mail.

<u>Contact Information:</u>
Protocol Registrar PHONE (336) 713-6767
Protocol Registrar FAX (336) 713-6772
Protocol Registrar E-MAIL (registra@wakehealth.edu)

*Protocol Registration is open from 8:30 AM - 4:00 PM, Monday-Friday.

4. Fax/e-mail ALL eligibility source documents with registration. Patients **will not** be registered without all required supporting documents.

Note: If labs were performed at an outside institution, provide a printout of the results. Ensure that the most recent lab values are sent.

To complete the registration process, the Registrar will:

- assign a patient study number
- assign the patient a dose
- register the patient on the study

5.0 Treatment Plan

Patients recruited at 500 mg/m² will receive this dose for all treatment phases.

Salvage Induction Cycle 1:

The dose of CPI-613 will be 500 mg/m²/day or 1,500 mg/m²/day given over 2 hours on days 1-5. Immediately following CPI-613 dosing 50 ml of D5W will be run through the central line. Cytarabine will be given following CPI-613 at 3gm/m2 for age <60 or 1.5 gm/m2 for age >60 in 500 mL NS over 3 hours every 12 hours for 5 doses starting on day 3. Mitoxantrone will be given at 6mg/m2 daily for 3 doses given in 50 mL NS over 15 minutes after 1st, 3rd and 5th doses of cytarabine.

Dose modifications for cytarabine and mitoxantrone are as follows (Please see section 6 for CPI-613 dose modifications):

Hold Cytarabine and notify physician if:

- Bilirubin 1.5-3: Consider Decrease dose by 25%.
- Bilirubin greater than 3: Consider Decrease dose by 50%.
- Cr Cl less than 60 mL/min: Consider dose reduction.
- Hold mitoxantrone and notify physician if:
- Bilirubin greater than 3: Consider Decrease dose by 25%.

All dose modifications are at the discretion of the treating physician.

Salvage Induction Cycle 2 (Optional, at the discretion of the treating physician):

A repeat of the initial cycle as outlined above or at the discretion of the treating physician an abbreviated second cycle can be given. In the abbreviated course CPI-613 is given as above on days 1-3, cytarabine given as above for 3 doses starting on day 2, mitoxantrone given as above after the first and third cytarabine doses.

Salvage Consolidation:

All responding patients are eligible for consolidation therapy with up to 2 cycles of the abbreviated course of CPI-613 at 500 mg/m²/day or 1,500 mg/m²/day, high dose cytarabine and mitoxantrone. Responding patients can be removed from trial at any time

to receive a stem cell transplant at the discretion of the treating physician. Patients can move to maintenance following 1, 2 or no cycles of consolidation at the discretion of the treating physician.

Maintenance Therapy:

Any patient that has completed all planned consolidation therapy (i.e. 0, 1 or 2 cycles) and refuses or is not eligible for a stem cell transplant may receive maintenance therapy with CPI-613. The dose for the maintenance phase of treatment in this study is 2,500 mg/m^2 except for patients who received 500 mg/m^2 at induction. Patients receiving 500 mg/m^2 at induction will continue with this dose during maintenance therapy. Patients may continue on maintenance therapy until evidence of disease progression, availability of stem cell transplant or occurrence of intolerable side effects.

5.1 Table of Study-Related Interventions

Assessments	Pre-Enrollment Screening	Salvage Induction Cycle 1							
		**Day1**	**Day2**	**Day3**	**Day4**	**Day5**	**Day6**	**Day7**	**Day 8**
Treatment with CPI-613		√	√	√	√	√			
Treatment with Cytarabine				√	√	√			
Treatment with Mitoxantrone				√	√	√			
Medical history[7]	√								
Physical exam[7] and vital signs[1]	√[1]	√[4]	√[4]	√[4]	√[4]	√[4]			
Pregnancy test for woman of child-bearing potential	√								
TTE, MUGA or MRI	√								
ECOG performance status	√								
Evaluation of symptoms & medications	√								
Clinical chemistry[2], hematology & coagulation[7,11]	√	√[5,6]	√[5,6]	√[5,6]	√[5,6]	√[5,6]	√[5,6]	√[5,6]	√[5,6]
Creatinine, BUN		√[10]	√[10]	√[10]	√[10]	√[10]	√[10]	√[10]	√[10]
Anti-Tumor Efficacy (i.e., imaging for granulocytic sarcoma, bone marrow biopsy for AML)[3.7]	Obtained within 2 weeks prior to the first dose.	Obtained on day 14 of cycle 1.							
Optional blood, urine and plasma samples, as well as bone marrow via biopsy[9] for possible testing of biomarkers, predictors of biological responses, toxicity, genotype vs. drug response relationship, etc.	Obtained within 2-4 weeks prior to the first dose.	Blood sample to be obtained prior to and following the dose of CPI-613 at 2, 4, and 6 hours from end of CPI-613 infusion on day 1 and bone marrow sample to be obtained on day 14 of Cycle 1. Bone marrow samples may be collected +/- 1-2 days if falls on a weekend or holiday. Optional urine samples will be collected from the first void after the first infusion of CPI-613. Details of the collection will be recorded on Appendix G.							

[1] Height, weight are also determined during pre-enrollment screening.
[2] Renal function will be assessed utilizing the Cockcroft-Gault formula.
[3] The Response Criteria will be assessed by standard criteria (Blood. 2010;115:453-474).
[4] Vital signs will be performed immediately after CPI-613 administration and the patient only re-examined if clinically indicated.
[5] These tests are performed within 24 hrs prior to dosing. Only the results of the creatinine are needed before CPI-613 is dosed.
[6] For Day 1 of Treatment Cycle 1, the pre-enrollment test results can be used for all labs except creatinine which must be rechecked 24 hours prior to dosing.
[7] The frequency of assessment beyond Cycle 1 is:
- For medical history, physical exam and vital signs, ECOG PS, evaluation of symptoms and medications, they are assessed within 5 days of the next cycle doses.
- Clinical chemistry and hematology are assessed on day 5 of each cycle (or day 3 if short dosing cycle is given).

[9] Bone Marrow via biopsy will only be collected if deemed appropriate by physician
[10] Creatinine and BUN to be checked within 24 hours of every dose and at 24, 48 and 72 hours post last dose of CPI-613, mitoxantrone, and cytarabine (i.e., days 6, 7, and 8)
[11] Coagulation (PT/PTT) to be completed Day 1 and weekly during Cycle 1.

Assessments	Salvage Consolidation Cycle 1 and 2							
	Day1	Day2	Day3	Day4	Day5	Day6	Day7	Day 8
Treatment with CPI-613	√	√	√					
Treatment with Cytarabine		√	√					
Treatment with Mitoxantrone		√	√					
Medical history[7]	√[7]							
Physical exam[7] and vital signs[1]	√[4]	√[4]	√[4]					
Pregnancy test for woman of child-bearing potential	√							
TTE, MUGA or MRI	√[11]							
ECOG performance status	√							
Evaluation of symptoms & medications	√							
Clinical chemistry[2] and hematology[7,11]	√[5]	√[5]	√[5]					
Creatinine, BUN	√	√	√					
Anti-Tumor Efficacy (i.e., imaging for granulocytic sarcoma, bone marrow biopsy for AML)[3.7]	Obtained upon completion of all planned consolidation therapy.							
Optional blood and plasma samples, as well as bone marrow via biopsy[9] for possible testing of biomarkers, predictors of biological responses, toxicity, genotype vs. drug response relationship, etc.	Blood sample to be obtained prior to and following the dose of CPI-613 at 2, 4, and 6 hours from end of CPI-613 infusion on day 1 and bone marrow sample to be obtained at the end of planned consolidation therapy. Bone marrow samples may be collected +/- 1-2 days if falls on a weekend or holiday.							

[1] Height, weight are determined during pre-enrollment screening.
[2] Renal function will be assessed utilizing the Cockcroft-Gault formula.
[3] The Response Criteria will be assessed by standard criteria (Blood. 2010;115:453-474).
[4] Vital signs will be performed immediately after CPI-613 administration and the patient only re-examined if clinically indicated.
[5] These tests are performed within 24 hrs prior to dosing. Only the results of the creatinine are needed before CPI-613 is dosed.
[7] The frequency of assessment is:
- For medical history, physical exam and vital signs, ECOG PS, evaluation of symptoms and medications, they are assessed within 5 days of the next cycle doses.

[9] Bone Marrow via biopsy will only be collected if deemed appropriate by physician
[10] Creatinine and BUN to be checked within 24 hours of every dose of CPI-613
[11] TTE/MUGA/Cardiac MRI can be obtained within 2 weeks of consolidation as long as no other cardiac toxins have been administered. Prior to cycle 2 of consolidation TTE/MUGA/Cardiac MRI is at the discretion of the treating physician

Assessments	Maintenance Therapy							
	Day1	Day2	Day3	Day4	Day5	Day6	Day7	Day 8
Treatment with CPI-613	√	√	√	√	√			
Medical history[7]	√[7]							
Physical exam[7] and vital signs[1]	√[4]	√[4]	√[4]	√[4]	√[4]			
Pregnancy test for woman of child-bearing potential	√							
ECOG performance status	√[7]							
Evaluation of symptoms & medications	√[7]							
Clinical chemistry[2] and hematology[7,11]	√[5]	√[5]	√[5]	√[5]	√[5]			
Creatinine, BUN	√	√	√	√	√			
Anti-Tumor Efficacy (i.e., imaging for granulocytic sarcoma, bone marrow biopsy for AML)[3.7]	Obtained upon completion of every fourth cycle.							

Optional blood and plasma samples, as well as bone marrow via biopsy[9] for possible testing of biomarkers, predictors of biological responses, toxicity, genotype vs. drug response relationship, etc.	Blood sample to be obtained prior to and immediately following the dose of CPI-613 on day 1and bone marrow sample to be obtained at the end of planned maintenance therapy. Bone marrow samples may be collected +/- 1-2 days if falls on a weekend or holiday.

[1] Height, weight are determined during pre-enrollment screening.
[2] Renal function will be assessed utilizing the Cockcroft-Gault formula.
[3] The Response Criteria will be assessed by standard criteria (Blood. 2010;115:453-474).
[4] Vital signs will be performed immediately after CPI-613 administration and the patient only re-examined if clinically indicated.
[5] These tests are performed within 24 hrs prior to dosing. Only the results of the creatinine are needed before CPI-613 is dosed.
[7] The frequency of assessment for cycles after cycle 1 is:
- For medical history, physical exam and vital signs, ECOG PS, evaluation of symptoms and medications, they are assessed within 7 days of the next cycle doses.

[9] Bone Marrow via biopsy will only be collected if deemed appropriate by physician.
[10] Creatinine and BUN to be checked within 24 hours of every dose of CPI-613.

5.2 Pre-Enrollment Medical Screening

Informed consent must be obtained prior to pre-enrollment medical screening. Investigators who are listed on US “Food and Drug Administration (FDA) Form 1572” or Canadian “Clinical Trial Site Information Form” are authorized to obtain informed consent. Pre-enrollment medical screening is used to determine the eligibility of each candidate. All enrollment evaluations must be performed within the time frame listed below, prior to CPI-613 treatment and include:

Within 2 weeks:
- Bone marrow biopsy or cytologic evidence of relapsed or refractory AML or granulocytic sarcoma
- Optional blood, plasma and bone marrow biopsy sample for biomarker evaluation
- a complete medical history
- physical exam, including vital signs, height, and weight
- ECOG PS
- baseline evaluation of symptoms and medications
- clinical chemistry (including renal function), hematology and coagulation (see Section 5.6 for specifics)

Within 1 week:
- a pregnancy test for women of child-bearing potential (performed within 1 week prior to CPI-613 treatment)

5.3 CPI-613 Dosing

This is a Pilot trial to determine the feasibility of CPI-613 given with high dose cytarabine and mitoxantrone in salvage induction, consolidation and maintenance. This is an open-label study, and investigators and subjects are not blinded to the treatment. The reason this is an open-label study is because this is a feasibility trial, and the investigators need to determine whether this regimen is feasible to be tested in larger clinical trials. The assignment of patients will not be randomized, since this is a feasibility trial. The doses of CPI-613 for the induction and consolidation phases of treatment in this study are 500 mg/m^2 or 1,500 mg/m^2 with 20 patients in each cohort for both 500 and 1,500 the study has already accrued 17 patients at 2,000 mg/m^2 cohort; the dose for the maintenance phase of treatment in this study is 2,500 mg/m^2 except for patients who received 500mg/m^2

at induction. Patients receiving 500mg/m^2 at induction will continue with this dose for all phases of therapy including maintenance.

CPI-613 must be diluted from 50 mg/mL to 12.5 mg/mL with D5W (i.e., 1 portion of CPI-613 diluted with 3 portions of D5W) and must be administered IV at a rate of ~0.5 mL/min via a central venous catheter with D5W running at a rate of about 125-150 mL/hr. Please refer to Section 5.4 for methods of administering CPI-613.

5.4 CPI-613 Administration Guidelines

Since information on the use of CPI-613 in humans is limited, investigators should be aware of potential AEs and toxicities observed in preclinical studies, as described below.

According to animal toxicology studies, IV administration of CPI-613 can induce reactions at and around the site of administration. These reactions are consistent with local inflammation, which include red/purple/black color, swelling, necrosis, warm to touch, etc. These local reactions occurred more severely when there was accidental leakage of CPI-613 into the perivascular space during IV administration. This is possibly because of low perfusion in the perivascular areas, causing prolonged exposure of local tissues to CPI-613. Therefore, care must be taken to avoid perivascular leakage of CP-613.

CPI-613 must be administered IV by infusion (not bolus), via a central venous catheter that is free flowing, with D5W running at a rate of about 125-150 mL/hr, and free of air in the dead space of the IV catheter. This is to minimize vascular irritation, inflammation and acute toxicity of CPI-613. Accidental co-administration of extra air that is present in the dead space of an IV catheter has been shown to induce excessive acute toxicity of CPI-613 according to animal studies (Study NCL-049).

CPI-613 must be diluted prior to administration, as described in Section 5.3. Dilution of CPI-613 prior to administration reduces the rates of delivery of CPI-613 to the body, thus minimizing the acute toxicity of CPI-613.

Accordingly, the following procedures in administering CPI-613 should be taken to minimize local reactions and acute toxicity of CPI-613:

A. Confirmation of the placement of the IV line to ensure that there is a lack of leakage of CPI-613 into the perivascular space.
B. Confirmation that the IV line is free flowing and with D5W running at a rate of about 125-150 mL/hr.
C. Confirmation that the IV line is free of air in the dead space.
D. If necessary, rotate the site of administration for different doses of CPI-613.
E. Dilute CPI-613 drug product with D5W, as instructed in the study protocol.

F. Administer CPI-613 by infusion, not bolus, as instructed in the study protocol.
G. Upon completion of administering CPI-613, “slowly” flush the IV line with ~10 mL of D5W to remove residual CPI-613.

Investigators should be aware of the following toxicity observed in animals treated with toxic doses of CPI-613:

1. CPI-613 at the toxicological doses of 30-35 mg/kg (180-210 mg/m²) induced dose-related incidence of mortality in rats. The cause of death in most rats was due to significant systemic inflammation, and might be related to an over enhancement in the inflammatory responses induced by CPI-613. Deaths due to significant systemic inflammation were also observed in minipigs treated with the minipig toxicological dose of 46.2 mg/kg (i.e., 1617 mg/m²) of CPI-613. Additionally, CPI-613 at 30 and 35 mg/kg (i.e., 180 and 210 mg/m², respectively) in minipigs increased reticulocyte levels, which appeared to be associated with hematopoietic cell proliferation of the spleen, and sternal bone marrow hyperplasia that were secondary to inflammation initiated at the administration site or port. Therefore, investigators should be aware of significant increases in reticulocyte levels, which can be used as an indication of severe inflammation.

2. CPI-613 at 42-55 mg/kg (i.e., 1470-1925 mg/m²) induced transient vomiting in minipigs.

3. CPI-613 at 42-46.2 mg/kg (1470-1617 mg/m², respectively) induced transient and reversible increases in troponin I and creatine kinase (CPK) isoenzyme. The transient and reversible nature of the troponin and CPK isoenzyme results were consistent with necropsy exam and histopathology, which indicated that there was no cardiac damage after the 3-week treatment of different doses of CPI-613.

5.5 General Concomitant Medication and Supportive Care Guidelines

Patients will receive standard prophylactic treatment for drug-related symptoms. Supportive treatments may include anti-emetic, anti-diarrhea, anti-allergic, anti-hypertensive medications, analgesics, antibiotics, allopurinol, and others such as blood products and bone marrow growth factors. The treating physician may utilize marrow growth factors, or blood or platelet transfusions at their discretion. For patients with an ANC ≥ 500/mm3 at the time of CPI-613 administration loperamide can be administered as a premedication at a dose of 2 to 4 mg PO 30 minutes prior to CPI-613.

5.6 Study Procedures – Assessment of Safety, Plasma Concentration, and Possible Anti-Tumor Efficacy

Safety Assessment: The safety of CPI-613 will be assessed based on:

- physical exams

- vital signs
- clinical pathology
    - clinical chemistry
    - renal function
    - hematology

For physical exams and vital signs, they are to be performed during screening (performed within 2 weeks prior to treatment with CPI-613) and after CPI-613 administration in Cycle 1. Beyond Cycle 1, physical exam and vital signs will be assessed within 5 days of the next cycle doses.

For clinical pathology (i.e., clinical chemistry, renal function, hematology and coagulation), they are to be performed during screening (performed within 2 weeks prior to treatment with CPI-613), and within 24 hrs prior to dosing. Creatinine results within the last 24 hours must be reviewed prior to CPI-613 dosing. For Day 1 of Treatment Cycle 1, pre-dose assessments of clinical pathology are not needed because the pre-enrollment results can be used. Beyond cycle 1, laboratory assessments will be assessed on at least day 1 and 5 of each cycle rather than prior to beginning each cycle.

For left ventricular ejection fraction assessments, they are evaluated during screening and prior to consolidation therapy. Additional assessments can be made at the discretion of the treating physician.

Anti-Tumor Efficacy – A Secondary Endpoint: Baseline anti-tumor efficacy (i.e., bone marrow biopsy) will be obtained within 2 weeks prior to the first dose of treatment cycle 1. Follow-up anti-tumor efficacy will be obtained on day 14 of cycle 1. In patients treated with additional treatment cycles beyond Cycle 1, anti-tumor efficacy will be assessed upon count recovery (defined as ANC>1000, freedom from RBC transfusions and platelets>100k) or day 42 from the first day of the cycle whichever comes first. Anti-tumor efficacy assessment will also be based on investigator and institutional routine monitoring of the disease progression of the patients, together with baseline evaluations performed prior to treatment with CPI-613. During maintenance therapy anti-tumor efficacy will be assessed after every 4th cycle.

Optional Sampling and "Banking" of Blood, Plasma, urine and Bone Marrow Biopsy Samples: Optional blood samples (5 mL), urine (5-10 ml) as well as bone marrow via biopsy (if deemed appropriate by physician), will be obtained and "banked" for possible testing of biomarkers, predictors of biological responses, toxicity, relationship between genotype and drug responses, etc. These samples will be obtained prior to treatment initiation, as well as prior to and following administration of CPI-613 during salvage Cycle 1. The specific procedures are outlined below.

<u>Whole Blood Sample</u>

- One (5 mL) Lavendar Top K EDTA Vacutainer Tube will be used.
- Do not centrifuge or freeze sample.

- Label sample and store at refrigerated temperature (2-8°C).
- Samples will be shipped on ice pack to the address shown at the bottom of this section.

Fresh Bone Marrow Biopsy for Banking of Tumor Cells

- Samples will be collected into a green top lithium heparin tube. Harvested fresh tumor specimens are placed on ice and shipped to the address below. (Note the tumor bank will Ficoll separate mononuclear cells and freeze aliquots in RPMI media containing 10% DMSO.)

Urine Samples

Urine samples will be collected from the first void after the first infusion of CPI-613 in the first induction cycle only. Details of the collection will be recorded on Appendix G.

- Approximately 5-10 ml of urine will be collected in a 15 mL conical tube
- The tube will placed at 4°C until placed into the Tumor Tissue Core Facility (see below)

All optional samples and specimens should be shipped to:

Wake Forest Cancer Center
Tumor Tissue Core Facility
Care of Dr. Greg Kucera
Hanes Building Rm 4049
Medical Center Blvd
Winston-Salem NC 27157

5.7 Specifics of Tests Performed During the Study

ECOG PS: The ECOG performance status (see Table below) will be used to classify patient's functional impairment. The higher the score, the worse the survival for most serious illnesses including AML.

ECOG Performance Scale (EPS)

Score	Definition
**0**	Fully active, able to carry on all pre-disease performance without restriction
**1**	Restricted in physically strenuous activity but ambulatory and able to carry out work of a light or sedentary nature, e.g., light house work, office work.
**2**	Ambulatory and capable of all self-care but unable to carry out any work activities. Up and about more than 50% of waking hours.
**3**	Capable of only limited self-care, confined to bed or chair more than 50% of waking hours.
**4**	Completely disabled. Cannot carry on any self-care. Totally confined to bed or chair.
**5**	Dead.

Lab Studies: Clinical chemistry assessed includes the following parameters. Renal function will be assessed utilizing the Cockcroft-Gault formula.

glucose	uric acid
creatinine	blood urea nitrogen (BUN)
total protein	AST/serum glutamic-oxaloacetic transaminase (SGOT)
albumin	ALT/serum glutamic-pyruvic transaminase (SGPT)
Na^+	alkaline phosphatase (ALP)
K^+	lactate dehydrogenase (LDH)
Cl^-	total bilirubin
Mg	
Ca^{+2}	
PO_4	
CO_2	

Hematology includes:

Complete blood count with differential

Coagulation includes:

INR	partial thromboplastin time

5.8 Future Research

Optional blood samples will be collected before and at various times after the 1st dose of CPI-613. Provisions should be made to record and report the actual clock times when samples are drawn. Whenever possible samples should be taken 5 minutes before and at completion of the infusion of CPI-613 on day 1 and at 2, 4 and 6 hours after the completion of the infusion. Blood (5 mL for each sample) should be obtained from an IV catheter that is not used for administration of CPI-613. The blood samples should be collected in 5-mL lavender top collection tubes containing K2- or K3-EDTA anticoagulant. Immediately after blood collection, gently invert tube 3 times to ensure proper mixing of blood and anticoagulant. Blood should then be immediately placed at 4 degrees Celsius. Additionally, several milliliters of urine from the first void following completion of the CPI-613 infusion on day one will be collected in consenting subjects. Once collected 5-10 ml of urine should be placed in a 15 ml conical tube and placed at 4 degrees Celsius until it can be sent to the tumor tissue core facility (see below).

When all samples have been collected, they should be sent to:

Wake Forest Cancer Center
Tumor Tissue Core Facility
Care of Dr. Greg Kucera
Hanes Building Rm 4049
Medical Center Blvd
Winston-Salem NC 27157
Email: gkucera@wakehealth.edu

5.9 Criteria for Removal from Study

Listed below are criteria for the discontinuation of the study. However, patients who fail to return for the follow-up visits will be contacted and queried as to the reason they have failed to complete the study with special attention to health status.

A. Patients have the right to withdraw from the study at any time for any reason.

B. The investigator has the right to withdraw patients from the study according to his/her discretion, if the investigator determines that continued participation is not in the patient's best interest. As an excessive rate of withdrawals can render the study not interpretable, unnecessary withdrawal of patients should be avoided. When a patient discontinues investigational treatment, the investigator should make every effort to contact the patient and to perform a final evaluation. The reason(s) for withdrawal must be recorded. Criteria for terminating subject's participation in the study are listed below:

   - Protocol defined disease progression significantly greater than expected
   - Unacceptable toxicity of the investigational product
   - Patient withdrawal of consent
   - Investigator's discretion
   - Undercurrent illness: a condition, injury, or disease unrelated to the intended disease for which the study is investigating, that renders continuing the treatment unsafe or regular follow-up impossible
   - General or specific changes in the patient's condition that renders the patient ineligible for further investigational treatment
   - Non-compliance with investigational treatment, protocol-required evaluations or follow-up visits
   - Termination of the clinical trial by the sponsor

6.0 Dosing Delays/Dose Modifications for CPI-613

The occurrence of Grade 1 toxicity does not generally require dose modification for subsequent doses for that patient. However, if Grade 2 non-hematologic, non-infectious toxicity develops attributed as at least probably related to CPI-613, treatment can resume only after the Grade 2 toxicity has been reduced to Grade 1 or below, and the dose level for subsequent doses for that patient will be reduced by 25% of the dose at which such Grade 2 toxicity occur. If Grade 3 or 4 non-hematologic, non-infectious toxicity develops, dosing of that patient will be withheld and the patient shall be monitored for recovery from, and reversibility of, such Grade 3 or 4 toxicity. To resume treatment for a patient who has had Grade 3 or 4 toxicity, the Grade 3 or 4 toxicity must be reduced to Grade 1 or below, and the dose level for subsequent doses for that patient will be reduced to 50% of the dose at which such Grade 3 or 4 toxicity occur. If the reduced dose of CPI-613 results in no toxicity, patients may be dose escalated to the prior dose at the discretion of the treating physician.

Dose adjustments during maintenance therapy will be done as described above with the addition that should patients experience hematologic toxicity ≥ Grade 2 attributed to CPI-613 the next dose of maintenance therapy must be held until the Grade 2 toxicity has been reduced to Grade 1 or below, and the dose level for subsequent doses for that patient will be reduced by 25% of the dose at which such Grade 2 toxicity occurred. If Grade 3 or 4 hematologic toxicity develops, dosing of that patient will be withheld and the patient shall be monitored for recovery from, and reversibility of, such Grade 3 or 4 hematologic toxicity. To resume treatment for a patient who has had Grade 3 or 4 hematologic toxicity, the Grade 3 or 4 hematologic toxicity must be reduced to Grade 1 or below, and the dose level for subsequent doses for that patient will be reduced to 50% of the dose at which such Grade 3 or 4 hematologic toxicity occurred. If the reduced dose of CPI-613 results in no toxicity, patients may be dose escalated to the prior dose at the discretion of the treating physician.

7.0 Adverse Events List and Reporting Requirements

Given the severe nature of the disease and treatment modalities associated with this patient population, any adverse event lower than grade 3 during a patient’s treatment AE evaluation period will not be part of data analysis or subject to expedited reporting.

7.1 Adverse Event Characteristics

- **CTCAE term (AE description) and grade:** The CTEP Active Version of the NCI Common Terminology Criteria for Adverse Events (CTCAE 4.0) will be utilized for AE reporting. The CTEP Active Version of the CTCAE is identified and located on the CTEP website at (http://ctep.cancer.gov/protocolDevelopment/electronic_applications/ctc.htm. All appropriate treatment areas should have access to a copy of the CTEP Active Version of CTCAE.

- **‘Expectedness’**: AEs can be ‘Unexpected’ or ‘Expected’ (see Section 7.1 above) for expedited reporting purposes only.

- **Attribution** of the AE:
    - Definite – The AE *is clearly related* to the study treatment.
    - Probable – The AE *is likely related* to the study treatment.
    - Possible – The AE *may be related* to the study treatment.
    - Unlikely – The AE *is doubtfully related* to the study treatment.
    - Unrelated – The AE *is clearly NOT related* to the study treatment.

List of Adverse Events to be Reported:
*Abdominal pain
*Creatinine
*Diarrhea
*Injection site Reaction
*Nausea
*Vomiting

All adverse events should be reported on the adverse event log and in ORIS regardless of whether they are on this list.
Asterisk (*) denotes expected Adverse Events.

All SAEs are required to be reported to Rafael Pharmaceuticals via the provided SAE Reporting Form. All completed forms must be sent to Claudia Moore at Rafael. The Comprehensive Cancer Center of Wake Forest University will submit any applicable SAEs to the FDA.

SAE reports must be submitted to Rafael by fax to the following address:

Department of Regulatory and Clinical Affairs
Rafael Pharmaceuticals, Inc.
25 Health Sciences Drive
Stony Brook, NY 11790
Telephone: 631-444-6868
Telefax: 631-794-2319

7.2 STRC SAE Reporting Requirements

The Safety and Toxicity Review Committee (STRC) is responsible for reviewing SAEs for CCCWFU Institutional studies as outlined in Appendix B. STRC currently requires that all unexpected grade 4 and all grade 5 SAE's on these trials be reported to them for review. This procedure is a part of the CCCWFU Data Safety Monitoring Plan that our institution has on file at the NCI. All CRM staff members assisting a PI in investigating, documenting and reporting an SAE qualifying for STRC reporting are responsible for informing a clinical member of the STRC committee as well as the entire committee via the email notification procedure of the occurrence of an SAE.

7.3 WFUHS IRB AE Reporting Requirements

Any unanticipated problems involving risks to subjects or others and adverse events shall be promptly reported to the IRB, according to institutional policy. Reporting to the IRB is required regardless of the funding source, study sponsor, or whether the event involves an investigational or marketed drug, biologic or device. Reportable events are not limited to physical injury, but include psychological, economic and social harm. Reportable events may arise as a result of drugs, biological agents, devices, procedures or other interventions, or as a result of questionnaires, surveys, observations or other interactions with research subjects.

All members of the research team are responsible for the appropriate reporting to the IRB and other applicable parties of unanticipated problems involving risk to subjects or others. The Principal Investigator, however, is ultimately responsible for ensuring the prompt reporting of unanticipated problems involving risk to subjects or others to the IRB. The Principal Investigator is also responsible for ensuring that all reported unanticipated risks to subjects and others which they receive are reviewed to determine whether the report represents a change in the risks and/or benefits to study participants, and whether any changes in the informed consent, protocol or other study-related documents are required.

Any unanticipated problems involving risks to subjects or others occurring at a site where the study has been approved by the WFUHS IRB (internal events) must be reported to the WFUHS IRB within 7 calendar days of the investigator or other members of the study team becoming aware of the event.
Any unanticipated problems involving risks to subjects or others occurring at another site conducting the same study that has been approved by the WFUHS IRB (external events) must be reported to the WFUHS IRB within 7 calendar days of the investigator or other members of the study team becoming aware of the event.

Any event, incident, experience, or outcome that alters the risk versus potential benefit of the research and as a result warrants a substantive change in the research protocol or informed consent process/document in order to insure the safety, rights or welfare of research subjects.

8.0 Pharmaceutical Information

8.1 Pharmaceutical Accountability

Drug accountability logs will be maintained for all investigative agents used under this protocol. These logs shall record quantities of study drug received and quantities dispensed to patients, including lot number, date dispensed, patient identifier number, patient initials, protocol number, dose, quantity returned, balance remaining, and the initials of the person dispensing the medication.

8.2 Study Drug

Various Names of Investigational Drug

Chemical Name:	6,8-bis-benzylsulfanyloctanoic acid
Alternative Name:	6,8-bis(benzylthio)octanoic acid; or 6,8-bis-benzylsulfonyloctanoic acid
Chemical Abstract Society (CAS) No.:	95809-78-2
Pharmacologic Class:	altered energy metabolism-directed (AEMD) compound
Code Name:	CPI-613
Trade Name:	Bylantra (tentative)
Generic Name:	None

Description of CPI-613 Drug Product

CPI-613 is provided in 10-mL amber glass vials. Each 10-mL vial contains 10 mL of CPI-613 at a concentration of 50 mg/mL (in 1M triethanolamine [TEA, also called trolamine]), which is equivalent to 500 mg of CPI-613. The drug product of CPI-613 is a clear and colorless solution that is free of any particulate matter.

Handling of CPI-613

CPI-613 is an investigational drug and the toxicity in humans is not fully understood. All necessary precautions in handling potentially toxic chemicals must be strictly adhered to. Gloves and protective clothing must be worn when handling CPI-613. Avoid contact by all modes of exposure. If the solution contacts the skin, it must be washed immediately and thoroughly with soap and water. If the solution comes in contact with mucous membranes, the membranes must be flushed thoroughly with water. Spills should be picked up with absorbent material and the area must be washed at least 3 times with ethyl alcohol followed by water.

CPI-613 drug product is slightly photosensitive (Study# PHO-001). Therefore, after removal of CPI-613 drug product from the amber vials, CPI-613 drug product should be protected from excessive light before administration to patients.

Storage of CPI-613

CPI-613 should be stored under refrigeration, at 2°-8°C (36°-46°F), except when being prepared for administration.

If CPI-613 is to be transferred from one storage area to another, or is to be prepared for dosing, care must be taken to maintain appropriate product temperature.

UNDER NO CIRCUMSTANCES SHOULD CPI-613 BE FROZEN.

IV Infusion Sets, Syringes and IV Bags to be Used for Administration of CPI-613

CPI-613 must be administered IV by infusion, via a central IV catheter with D5W running at a rate of about 125-150 mL/hr. Subsequent sections describe the appropriate types of IV catheters, IV bags, syringes and clinical solutions that can be used in mixing and administering CPI-613 to patients.

Leaching of Diethylhexyl Phthalate (DEHP): CPI-613 can cause leaching of DEHP from IV infusion sets and IV bags (Study COM-003). Therefore, DEHP-containing IV infusion sets, IV bags or syringes should not be used in mixing or administration of CPI-613. Examples of syringes and extension set

for syringe pump that do not contains DEHP (and therefore can be used in the administration of CPI-613) are:

Syringes: Kendall Monoject syringes, all mono-ject syringes are DEHP free.

Extension Set for Syringe Pump Use: All extension sets from MED-RX do not contains DEHP.

<u>IV Infusion Sets</u>: A compatibility study has been conducted showing that CPI-613 is compatible with 4 commonly used IV infusion sets (Study# COM-001). Therefore, these 4 types of IV infusion sets, and IV infusion sets that are made with the same materials, can be used to administrate CPI-613. These IV infusion sets are:

PVC material - ADDitIV® Primary IV Set with Universal Spike, Backcheck Valve, 2 Injection Sites, DEHP-Free and Latex-Free, 15 drops/mL, REF V14453, B Braun Medical Inc.

Latex material - Interlink® System Secondary Medication Set, 10 drops/mL, 2C7451, Baxter Healthcare Corporation

PVC material - Surshield™ Safety Winged Infusion Set, 0.19 mL Volume, Latex-Free, DEHP-Free, SV*S25BLS, Terumo Medical Products Hangzhou Co. Ltd.

Polyethylene material - Interlink® System Paclitaxel Set by Baxter HealthCare, Non DEHP: Polyethylene tubing with a 0.22 microfilter Item # 2C7558 10 drops/mL

<u>Syringes</u>: Compatibility studies (Studies# COM-001 and COM-002) have shown that CPI-613 drug product (50 mg/mL), and drug product diluted with D5W to various concentrations (1.6-25 mg/mL) are compatible with various types of syringes, as listed below. Therefore, any of these types of syringes, and syringes that are made with the same materials, can be used to administer CPI-613. Also, glass syringes can also be used, since glass (such as glass containers) is compatible with CPI-613 drug product.

Norm-Ject, polythelyene barrel, polyethylene plunger, latex free (Henke Sass Wolf GMBH) syringes
Becton Dickinson syringes
Terumo syringes
Monoject syringes
Glass syringes

<u>Reconstitution and Administration of CPI-613</u>

CPI-613 must be diluted from 50 mg/mL to 12.5 mg/mL with 5% Dextrose Water or D5W (i.e., 1 portion of CPI-613 diluted with 3 portions of D5W) prior to administration. The diluted drug product should be visually inspected for clarity.

If haziness or precipitate is observed, do not use the diluted drug product for dosing. After dilution with sterile D5W, the solution is clear and has a pH of 8.4-8.8. The diluted CPI-613 drug product has been found to be stable for 24 hrs at room temperature and refrigeration temperature (Studies STA-010).

CPI-613 must be administered IV, via a central venous catheter that is free flowing, free of air in the dead space, and with D5W running at a rate of about 125-150 mL/hr. This is to minimize vascular irritation, inflammation and acute toxicity of CPI-613 (Study NCL-049). Accidental co-administration of extra air in the dead space of IV catheters during administration of CPI-613 has demonstrated the potential to induce acute toxicity of CPI-613 according to animal studies (Study NCL-049). Also, accidental leakage of CPI-613 into the perivascular space during IV administration, which prolongs exposure of perivascular tissue to CPI-613, can induce significant local inflammation according to animal studies (Studies NCL-027 and NCL-030).

CPI-613 must not be administered as a bolus, but by infusion via a central venous catheter, over a total of 2 hours. All doses of CPI-613 will be administered over 2 hours to simplify PK/PD analysis. The infusion will also minimize potential acute toxicity of CPI-613, according to animal studies (Study NCL-049). The following precautions must be taken when administering CPI-613:

A. Confirmation of the placement of the IV line to ensure a lack of leakage of CPI-613 into the perivascular space.
B. Confirmation that the IV line is free flowing, and with D5W running at a rate of about 125-150 mL/hr.
C. Confirmation that the IV line is free of dead air space.
D. Rotate the site of administration for different doses of CPI-613.
E. Dilute CPI-613 drug product with D5W, as instructed in the study protocol.
F. Administer CPI-613 by infusion, not as a bolus.
G. After administration of CPI-613, “slowly” flush the IV line with ~10 mL of D5W to remove residual CPI-613.

Request for CPI-613

CPI-613 must be requested from Rafael by the Principal Investigator (or authorized designees). CPI-613 may not be used outside the scope of this protocol, nor can it be transferred or licensed to any party not participating in this clinical study. Rafael policy requires that CPI-613 be shipped directly to the institution where the patient is to be treated. Rafael does not permit the transfer of CPI-613 between institutions (unless with prior written approval from Rafael). Requests must be submitted to Rafael by fax or email to the following address:

Department of Regulatory and Clinical Affairs
Rafael Pharmaceuticals, Inc.
1 Duncan Dr

Cranbury NJ 08512
Telephone: 631-444-6868
Telefax: 631-794-2319
Email: sanjeev@Rafaelpharma.com

The following information must be provided in the request of CPI-613 from Rafael:

- Names of the principal investigator and the requestor (if different)
- Name of the study site
- Name of the pharmacist responsible for receiving and storing CPI-613
- Name of the person and address where CPI-613 is to be shipped to
- Amount (# vials) requested
- Date of request
- Date shipment expected
- Study Protocol (title and protocol#) for which the requested CPI-613 is to be used

Procurement of Investigational Drug

Relevant regulations require investigators to establish a record of the receipt, use and disposition of all investigational products. Investigators may delegate responsibility of drug ordering, storage, accountability and preparation to their designees.

The investigator, or the designee, will be responsible for dispensing and accounting of CPI-613 provided by Rafael and for exercising accepted medical and pharmacy practices.

Records of inventory, dispensation and disposition (vials received, source and dates) must be maintained. In addition, all doses dispensed should be accounted for by recording the date, study number and name, patient identification, patient initials, patient medical record number and balance forward. These records must be maintained and kept at the study site, and will be reviewed by Rafael, or its designee, during periodic monitoring visits.

Disposal of CPI-613

The following procedures are to be taken in disposal of CPI-613:

- During the study, store the used CPI-613 vials (which must be separate from the unused CPI-613 vials) at room temperature in an access-limited area. Alternatively, destroy the used CPI-613 vials according to institutional policy after documentation of the number of used CPI-613 vials and remaining volume in each used vial.

- At the end of the study, deface the label (both used and unused vials) with a permanent marking pen.
- For used CPI-613 vials (if not already destroyed according to institutional policy), after documentation of the number of used CPI-613 units and remaining volume in each container, the used containers should be destroyed at the site according to the institutional procedures for destroying toxic chemicals. A certificate documenting the destruction of used vials must be kept on file.
- All unused CPI-613 vials must be returned to Rafael at the address shown below. (Note: The return of the CPI-613 to Rafael, as well as the quantity returned, must be documented.)
  Department of Regulatory and Clinical Affairs
  Rafael Pharmaceuticals, Inc.
  25 Health Sciences Drive
  Stony Brook, NY 11790
  Telephone: 631-444-6868
  Telefax: 631-794-2319
  Email: king@Rafaelpharma.com

9.0 Measurement of Effect

9.1 Antitumor Effect

Although response is not the primary endpoint of this trial, patients with measurable disease will be assessed by standard criteria for AML (*Blood.* 2010;115:453-474). Peripheral blood (PB) cell counts and samples of bone marrow aspirates will be assessed for changes in disease status. Hematologic improvement (HI) requires improvement in one of the three hematopoietic lineages compared with baseline measurements, and stable disease (SD) requires the absence of objective disease progression for at least 2 months. Relevant responses have to be confirmed by a repeat measurement at least 1 wk later. Assessments will be done as described above.

9.1.1 Definitions

Evaluable for toxicity. All patients will be evaluable for toxicity from the time of their first treatment with CPI-613.

Evaluable for objective response. Only those patients who have measurable disease present at baseline, have received at least one cycle of therapy, and have had their disease re-evaluated will be considered evaluable for response. These patients will have their response classified according to the definitions stated above. (Note: Patients who exhibit objective disease progression prior to the end of cycle 1 will also be considered evaluable.)

Overall Survival (OS): Time from enrollment on trial to death from any cause.

10.0 Statistical Considerations

This is a pilot trial investigating three doses of CPI-613 (500, 1500, and 2,000 mg/m^2. We plan to accrue between 10 and 15 patients per year for a total of 67 patients (20 at 500, 1,500 mg/m^2 and 3 additional patients at 2,000 mg/m^2). Of note 17 patients at 2,000 mg/m^2 and 7 patients at 2,500 mg/m^2 were already accrued.

Analysis Plan:

The primary objective of determining the feasibility of administering CPI-613 in combination with high dose cytarabine and mitoxantrone during induction, consolidation and maintenance therapies will be completed by determining the percentage of patients eligible for maintenance therapy who complete at least 3 cycles. If ≥50% of eligible patients complete 3 cycles of maintenance therapy we will consider this regimen feasible for future study. Given an estimated response rate of 50% and a 25% attrition to stem cell transplant, if we enroll 60 patients we should be able to assess feasibility of maintenance in approximately 22 patients. With 22 evaluable patients, a two-sided 95.0% confidence interval using the large sample normal approximation will extend 22% in either direction from the observed value when the expected value is 50%. Within a dose we should be able to estimate feasibility in 7 patients with a 95% confidence interval extending 36% in either direction.

Participants will also be followed and analyzed for secondary outcomes of response rate and overall survival both overall and by dose. Participants will be monitored for survival through routine follow-up visits. Confidence intervals will be calculated around the estimates of the response rate (CR and CRi). Assuming a response rate of 0.5, with 60 participants, we would be able to create 95 percent confidence intervals with a 0.13 margin of error (0.37, 0.63). We will use Kaplan-Meier estimation to analyze overall survival. The frequency of toxicities experienced by the participants will be presented by type and grade in an effort to monitor and report safety of the treatment.

In exploratory analysis we plan to compare both complete response and early mortality (death within 60 days of beginning of treatment) to the observed rates in a historical cohort of subjects (CCCWFU 22111), both overall and by dose. In the historical cohort the response rate was estimated to be 41%. Using a one-sided chi-square test, a 0.05 significance level, null hypothesis of 41%, and assuming 70% power, we would be able to show significant improvement in response if we are able to achieve a response rate of 56% or higher. Early mortality in the historical cohort was estimated to be 29% at 60 days. We will use a one-sided exact test for a single proportion, a 0.05 significance level, a null hypothesis of 29%, and 70% power. To show a significant decrease in early mortality in this study we would need to observe a 60 day mortality rate of 16.8% or lower.

Supplementary References

1. Baggetto LG. 1992. Deviant energetic metabolism of glycolytic cancer cells. *Biochimie* **74**:959-974.

2. DeGeorge JJ, Ahn C-H, et. al. 1998. Regulatory considerations for preclinical development of anticancer drugs. Cancer Chemother. Pharmacol. **41**:173-185.

3. Lutes RA. 2006. A Dose-Escalation Study of CPI-613 in a Single Patient with Metastatic Breast Cancer - Treatment Summary.

4. Lutes RA. 2008. A Dose-Escalation Study of CPI-613 in a Single Patient with Metastatic Breast Cancer - Treatment Summary.

5. Therasse P, Arbuck S, Eisenhauer EA, et al. 2000. New guidelines to evaluate the response to treatment in solid tumours. J Natl Cancer Inst **92**:205–216.

6. Strevel EL, Ing DJ, Siu LL. 2007. Molecularly targeted oncology therapeutics and prolongation of the QT interval. J Clin Oncoly **25**:3362–3371.

7. Study COM-001. 2006. Compatibility Testing of CPI-613 Drug Product - Infusion Sets.

8. Study COM-002. Bhasin R. 2008. Compatibility Testing of CPI-613 Drug Product Dosing Solutions with Different Types of Syringes.

9. Study COM-003. Gupta D. 2009. Investigation of the Release Behavior of Diethylhexyl Phthalate (DEHP) from the Polyvinyl-Chloride Containing Infusion Sets and Bags for Intravenous Administration by CPI-613 Drug Product.

10. Study CL-CPI613-002. Ongoing. An Open Label, Dose-Escalation Study to Evaluate Safety, Tolerability, Maximum Tolerated Dose (MTD), Efficacy, and Pharmacokinetics (PKs) of CPI-613 Given Twice Weekly for Three Consecutive Weeks in Cancer Patients.

11. Study NCL-027. Seng J. 2006. Escalating Dose Toxicology Study of Intravenously Administered CPI-613 to Miniature Pigs.

12. Study NCL-030. Seng J. 2006. An Acute Toxicity Study of CPI-613 Administered Via the intravenous (Slow Bolus) Route to Mice.

13. Study NCL-044. Seng J. 2007. A 21-Day Toxicokinetic Study of CPI-613 Administered Twice Weekly via the Intravenous Route to Sprague-Dawley Rats with a 14-Day Recovery Period.

14. Study NCL-045. Seng J. 2008. Evalutation of the Toxicity Potential of CPI-613, Given Twice Weekly for 3 Weeks by Intravenous Administration, in Hanford Minipigs with a 14-Day Recovery Period.

15. Study NCL-049. Moore C, Karnik S, Lee K. 2007. Preliminary Studies of Comparative Toxicity of CPI-613 Administered Intravenously (IV) as Bolus Vs. Infusion, Effects of Air in Dead Space of Butterfly IV Infusion Set on Toxicity of CPI-613, and Acute Effects of CPI-613 on Clinical Chemistry in Rats.

16. Study PHO-001 (Covance Study# 7769-101). Potts B. 2007. Photostability Testing of CPI-613 Drug Product.

17. Study STA-004. (CR Study# LRH00017SX). McFarlene J. 2006. Long Term Matrix Stability Assessment of CPI-613 in K2 EDTA and K3 EDTA Human Plasma.

18. Study STA-010. Bhasin R. 2007. Stability of CPI-613 Injection dosing solutions after Dilution with 5% Dextrose (D5W).

19. Study VLD-002 (CR Study# LRH00016LX). McFarlene J. 2006. Validation of a High Performance Liquid Chromatographic-Mass Spectrometric Method for the Analysis of CPI-613 in K3 and K2 EDTA Human Plasma.

20. Yamada J, Tomiyama H, et. al. 2006. Elevated serum levels of alanine aminotransferase and gamma glutamyltransferase are markers of inflammation and oxidative stress independent of the metabolic syndrome. Atherosclerosis. 189(1):198-205.

Source Data for Supplementary Information:

Figure 3A

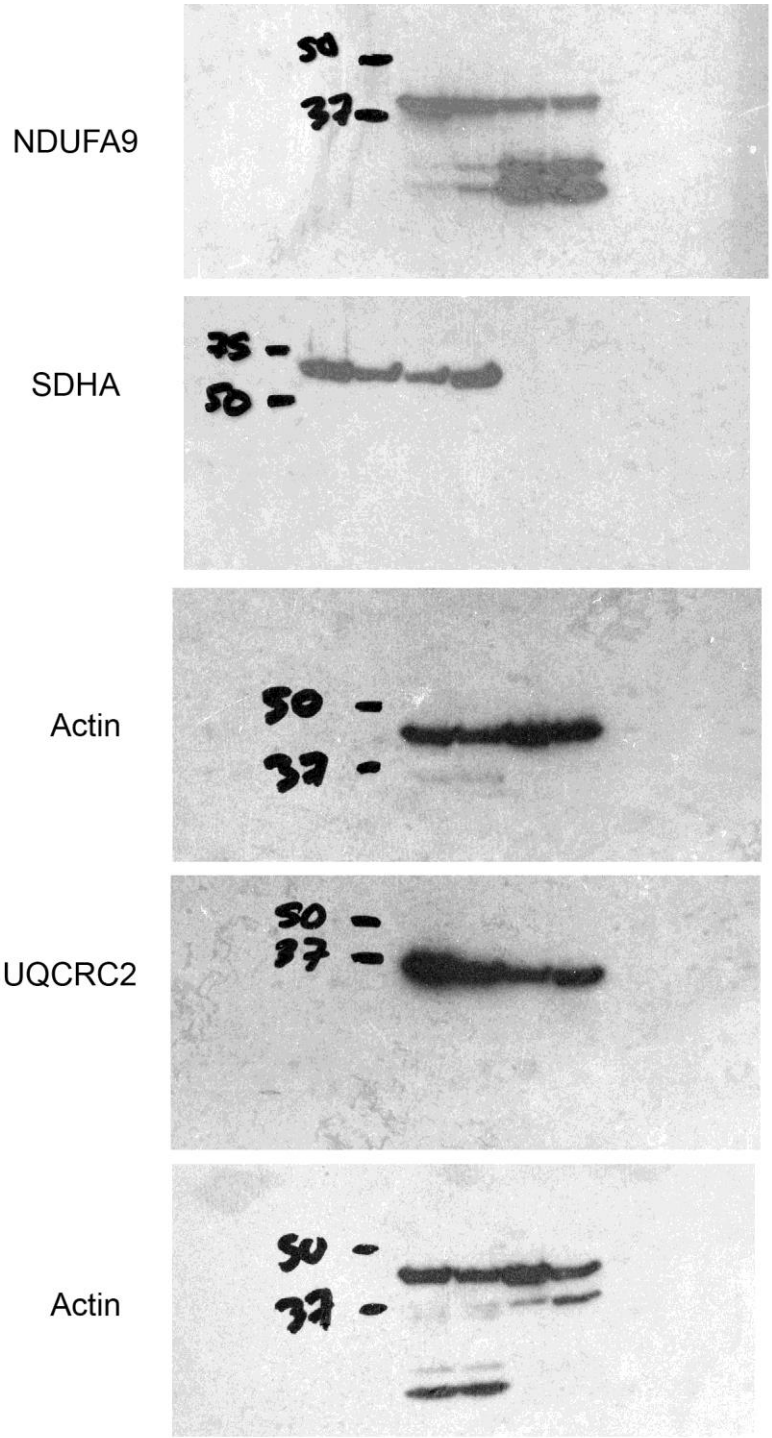

Figure 3B

NDUFA9

Y	O
1.181025	0.522139
1.118908	0.303254
1.135237	0.97816
2.327806	0.311589
1.422078	0.485638
0.752558	0.460316
0.717789	1.028298
0.962	0.354104
1.32716	0.557736
1.055564	0.313434
0.771062	1.645894
1.199248	0.260835
1.466021	
1.692284	
1.805355	
0.562815	
2.083487	
1.975647	

SDHA

Y	O
0.917545	0.512755
1.545912	0.447939
1.137206	0.869294
1.508381	0.544928
1.418775	0.759744
1.392635	0.623141
1.279952	1.770627
1.145204	0.660615
1.054997	0.863697
1.299002	0.722895
1.070986	1.763542
1.984301	0.580348
1.547559	
2.086274	
1.665218	
1.318489	
2.21989	
1.395189	

UQCRC2

Y	O
1.54784	0.727586

0.807486	0.416685
0.904469	0.381957
1.258121	0.388636
1.05187	0.335962
0.928141	0.312509
1.159822	1.076169
0.990197	1.288596
0.463357	0.844595
0.621268	0.540541
0.738411	0.538829
1.066873	0.379118
1.148291	
0.966915	
0.947036	
0.959154	
0.912796	

Figure 3C

Young

Erra	1.06	0.89	0.95	0.82	0.69	1.22	1.05	0.93	1.02
Nrf2	0.46	1.44	1.51	1.02	0.98				
Pcg1b	1.09	1.1	0.83	1.16	0.77	1.12	0.89	1.02	1.1
Tom20	0.75	1.34	0.99	0.95	1.15	0.91	0.99	0.97	1.04
Vdac1	0.9718	1.0242	1.0047	1.1793	0.8331	1.0178	1.12	1.36	0.89

Old

Erra	0.176	0.3129		0.0357	0.0184		0.0647	0.0231	0.0542
Nrf2	0.7059	0.2976	1.1448	0.4807	0.1303	0.4931			
Pcg1b	0.8085	0.2895	0.0336	0.7267	0.479		0.6735	0.7828	0.9604
Tom20	0.0108	0.009		0.0126	0.0044	0.0097	0.8584	0.9188	0.9974
Vdac1	0.0551	0.0006	0.0008	0.5992	0.3573	0.4978	0.0751	0.0327	0.0339

3D

Young	Old
575.07	251.53
237.63	254.2
312.49	127.97
291.74	307.83
250.74	243.94
335.56	178.04
262.29	175.92
226.72	272.28
335.93	134.67
262.4	128.02
289.15	212.48

258.89	294.41
294.33	129.63
244.61	244.05
540.85	158.84
272.73	161.14
437.04	116.55
884.58	181.28
626.94	233.89
209.96	169.27
140.7	134.62
181.44	93.4
259.28	55.28
262.15	595.93
820.95	166.69
165.5	106.46
193.04	112.24
329.45	282.26
237.58	164.6
154	175.55

Figure 3E Mito Content

Old	Young
0.777338	0.996957
0.773643	0.982196
0.782132	1.020846
0.793952	1.074818
0.834284	0.975526
0.927116	0.949656
0.73248	0.95776
0.714915	1.019748
0.763227	1.022492

Figure 3E MMP

Old	Young
0.897228	0.997797
0.909742	0.995794
0.929263	1.006409
0.89717	0.985888
0.900938	1.005033
0.908803	1.009079
0.782847	0.990002
0.81857	0.980758
0.841433	1.02924

Figure 3F Mito Content

Adolescent	Middle Aged
0.95566857	0.6856798
1.02999505	0.68359119
1.01433638	0.84266706
0.99141401	0.49028207
1.09958066	0.44475823
0.90900532	0.58266328
0.91133642	0.48930996
0.73830871	0.51624248
1.35035488	0.66901658

Figure 3F MMP

Adolescent	Middle Aged
1.00971432	0.89776241
0.98724191	0.92514425
1.00304377	0.97841218
1.13265473	0.84546408
0.78581141	0.88121139
1.08153386	0.79765337
0.97547272	0.79919349
1.003714	0.78219587
1.02081328	0.88421213

Figure 4A MitoSOX

Vehicle	Dev 50μM	Dev 100μM	Met 1mM	Met 2.5mM	Met 5mM	50-1	50-2.5	50-5	100-1	100-2.5	100-5
20280	70154.5	93041	18245	24898	34643	101211	113802	118503	119627	134121	146878
9020	44553	61562.5	13479	18150	23225	62830	80848	91724	91542.5	109942	136002
9617	68022	72760	13010	16498	20159	113298.5	125801.5	122946	128786.5	136418.5	158510

Figure 4B

veh	99.94485	105.3922	94.66299	96.19435	102.2765	101.5292	102.6308	103.8464	93.52282
Dev 50μM	60.93737	67.097	68.50035	78.06003	85.3173	87.32609	82.80024	79.93844	76.12452
Metformin 5mM	27.09009	26.95018	28.65464	26.12183	23.61571	27.03885	29.61527	24.81772	26.11959
NAC 1mM	105.3228	118.2808	125.1169	99.53175	111.8483	115.1623	108.884	104.9665	98.74382
50/5	10.66115	11.40449	11.7719	12.87895	14.66651	11.6109	14.0842	15.56561	13.4841
50/1	55.81458	64.49957	69.53415	60.69014	71.51174	70.98563	76.13289	72.65201	72.03164
5/1	19.52255	20.78051	22.41805	19.6269	16.9577	17.44813	18.61726	19.96032	16.85687
50/5/1	3.955262	4.670925	3.669969	7.255466	6.679297	6.113921	7.014962	6.655226	6.037432

Figure 4C

Vehicle	Dev 50μM	Met 5mM	NAC 1mM	50-5	5-1	50-5-1
0.982451	2.368101	1.148169	0.395665	3.383259	0.336012	3.856678
1.082074	2.313359	1.038839	0.301223	3.311315	0.360053	4.744404
0.935474	2.301865	1.107504	0.314336	3.264968	0.40523	4.044804
1.040654	2.518685	1.248064	0.815998	3.842496	1.257087	5.282192
0.972241	2.347652	1.316134	0.77697	3.039085	1.219633	4.23054
0.987105	2.281327	1.641044	0.901282	3.180978	1.22372	4.909615
1.070732	3.23899	1.088375	0.415479	5.624154	1.111166	5.133381
0.989599	3.283568	1.023565	0.315619	5.160959	1.121054	7.724008
0.939669	4.841243	1.075755	0.518367	5.437919	1.108796	5.635361

Figure 4D

Vehicle	Dev 50μM	FCCP 10μM	NAC 1mM	50+1	10+1
0.980235	2.574884	3.917342	0.591641	3.576182	0.989383
0.932834	2.54915	6.987959	0.415172	3.559687	0.979757
1.08693	2.524293	5.764237	0.516273	3.9436	0.965221
1.005487	2.341422	6.18112	0.899121	4.309237	1.231346
1.010204	2.524616	7.642071	0.886122	5.098066	1.059181
0.984309	2.568007	9.033567	0.719608	5.408518	1.160336
0.925651	3.262809	7.26095	1.053903	4.988524	2.042347
0.976806	2.973937	7.343624	0.871828	6.257718	2.03774
1.097543	2.712462	6.834613	0.910134	4.998465	2.12793

Figure 4E

Vehicle	Dev 50μM	AntA 2.5nM	50/2.5
104.0672	64.56657	72.98727	35.75464
95.06065	65.03963	72.401	27.68625
100.8721	63.53145	75.35778	30.96
95.25998	77.18556	87.48502	67.12208
101.8495	76.53141	85.09627	60.18244
102.8906	78.75953	84.29698	64.04315
94.06337	76.69312	87.61881	73.37847
105.6341	85.51254	89.42518	79.69029
100.3025	86.92017	88.52083	75.57608

Figure 4F

Vehicle	Dev 50μM	Para 10μM	50/10
98.29572	65.72085	68.13369	38.93871
95.73756	63.65058	69.18759	42.31774
105.9667	71.5197	73.78503	36.65723
98.71262	68.55446	87.49993	52.54382
100.9813	67.58542	82.85988	45.64882
100.3061	64.46743	76.73647	49.06007
94.06337	76.69312	95.87932	42.60882
105.6341	85.51254	91.66626	45.71751
100.3025	86.92017	80.5742	51.39124

Figure 4G

MFL2

Control	98.36368	100.2243	101.412	98.0114	105.7669	96.22166	102.7396	100.7168	96.54366	99.13346	99.31595	101.5506
Dox 10 nM	102.085	97.2948	100.7786	102.9034	94.55124	96.18189	80.92949	67.63799	78.88759	96.86094	84.16238	98.04885
Dev 50 μM	98.12615	98.48245	99.35339	94.51147	96.50007	96.85801	81.3602	82.12911	77.07859	78.41222	87.88449	89.29965
Dox+Dev	97.01768	87.63526	90.01056	96.53984	90.81267	83.85258	68.67808	61.67181	66.84994	71.11606	74.17707	73.89473
Dox+Dev +Met	34.62655	36.01214	39.77303	19.26289	28.25136	21.13218	18.84228	21.58832	21.20387	22.72243	22.7362	23.21343

RHRAS

Control	105.5632	102.0188	92.41799	100.5251	101.3734	98.10152	96.3608	100.143	103.4962
Dox 10 nM	94.31062	86.05185	86.56802	99.55568	95.19321	98.10152	72.65402	89.30335	72.92696
Dev 50 μM	89.21771	90.93829	96.40973	94.30456	98.86899	97.13209	96.94567	94.9961	89.42033
Dox+Dev	29.30718	39.7683	40.49094	9.196176	11.66016	11.82173	12.76319	24.22667	24.18768
Dox+Dev +Met	11.55081	9.658178	12.13581	6.328262	13.7606	13.39706	8.59111	11.78841	1.845594

Figure 4H

Days	Vehicle (n=5)	Dev (n=5)	Metformin (n=5)	Combo (n=5)
35	1			
35	1			
35	1			
35	1			
35	1			
36		1		
36		1		
36		1		
36		1		
36		1		
36			1	
36			1	
36			1	
36			1	
36			1	
28				1
30				1
41				1
41				1
41				1

Figure 5

Vehicle	Dev 50μM	Dev 100μM	Doxy 10μM	50-10	100-10
170240	213777	208240.5	191622.5	185646	191260.5
116242	156855	172613	154052	166568	190632
157999	189324.5	206879	151217	187554	210381

Figure 6A

ROSAKO

0	88.50834	102.9984	108.4933	99.71115	99.88446	100.4044	99.161	101.0317	99.80726
10	77.66905	91.10526	104.3156	98.42865	93.88793	94.23455	100.6916	98.4127	97.93651
15	78.6476	72.77631	87.45452	78.53264	71.3576	67.37146	78.68481	76.50794	72.69841
20	36.23134	33.10751	40.89826	22.06817	27.37146	26.67822	29.46712	27.29025	24.36508

PDH KO

0	88.32987	98.43011	113.24	77.6071	103.8295	118.5634	82.12074	105.9907	111.8885
10	61.32022	54.85152	63.58994	79.87884	77.54219	85.9801	77.94118	69.48916	61.22291
15	30.28182	31.24645	35.2752	37.04024	45.86759	31.91259	23.32817	24.48916	24.39628
20	16.03934	14.22357	14.73425	12.11597	14.45262	18.86629	10.2322	9.210526	8.653251

Figure 6B

Control	Dev 50μM	10	15	10/50	15/50
101.8354	92.98225	101.6915	52.92706	48.1406	13.23177
99.78407	91.39875	100.4319	52.02735	62.32006	12.00816
98.38052	96.04127	102.9511	46.34117	64.01152	9.452975
104.5659	111.1567	100.3573	42.74749	49.21916	9.634727
97.06194	107.6628	99.12652	35.95818	61.64637	10.74643
98.37215	111.1567	104.3674	35.1244	64.74325	10.58761
101.4495	112.9625	96.47985	68.07013	60.73992	26.36665
96.56267	117.7664	111.6786	61.65102	77.63666	16.4688
101.9879	119.2159	115.82	64.6328	71.42463	22.39094

Figure 6C

		Control			Dev 100μM	
Pt 19	102.1301	102.0445	95.82541	57.82617	49.52453	60.22252
Pt 137	100.0989	98.74089	101.1602	73.68887	69.69303	78.15297
Pt 146	96.59751	101.4274	101.9751	76.30705	85.3444	87.75934
Pt 210	101.7578	95.71794	102.5243	37.95606	36.76035	32.31477
		MDIVI 50μM			100/50	
Pt 19	66.01369	72.3469	65.27197	28.44237	22.08064	24.16318
Pt 137	68.27263	71.39438	70.11446	36.05619	40.95734	40.83247
Pt 146	42.6971	66.06224	57.97095	57.90871	64.08299	50.56432
Pt 210	93.08125	87.4093	89.61676	14.68574	9.075115	8.094021

Figure 7A

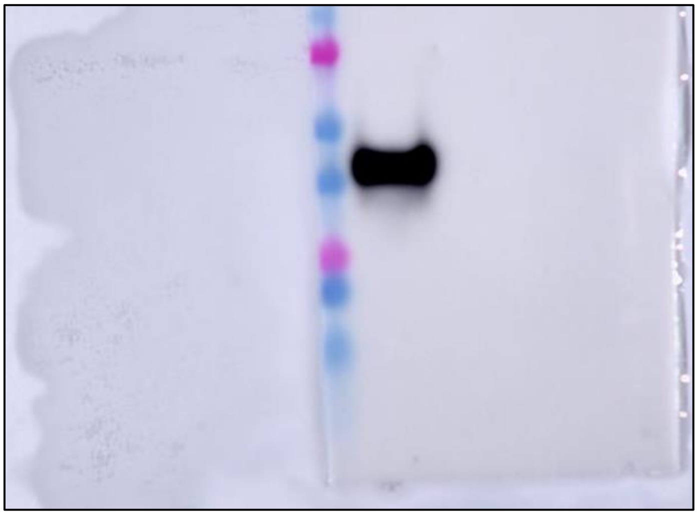

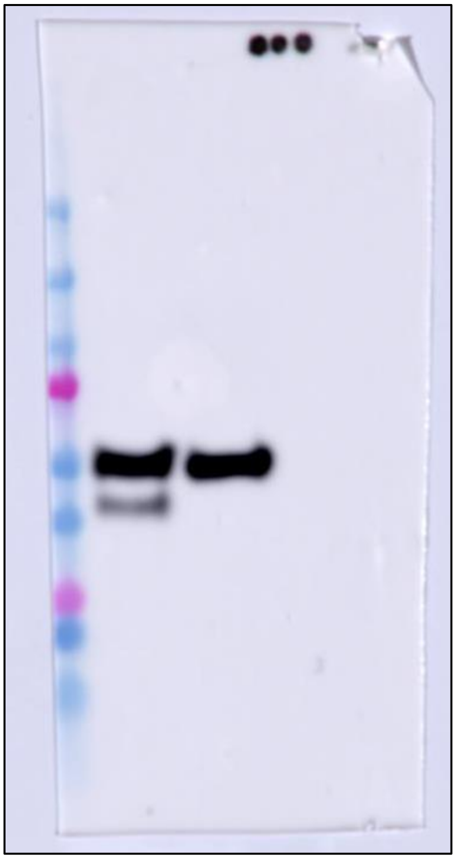

Figure 7B

K562 Control

0	101.5847	100.201	98.21429	100.8624	100.6109	98.52677	101.0013	100.2146	98.78412
30	100.7687	99.9527	100.4139	88.93281	95.22098	84.36939	96.63846	98.49803	100.2861
40	88.42242	86.96783	85.33586	58.96515	63.27704	57.20446	68.35141	94.81464	91.6319
50	46.06197	61.67219	59.8983	42.79554	44.7359	39.70535	39.77828	74.21624	75.25331

K562 PDH KO

0	99.81139	99.98821	100.2004	99.82047	100.2513	99.92819	99.87775	100.3545	99.76773
30	39.76188	41.88377	36.57904	65.31418	72.06463	76.26571	89.71883	92.57946	86.38142
40	31.4865	30.92066	28.51586	37.77379	36.62478	37.55835	70.57457	74.93888	66.46699
50	23.63551	23.0343	21.05387	23.37522	21.72352	22.98025	49.30318	41.52812	37.75061

Figure 7C

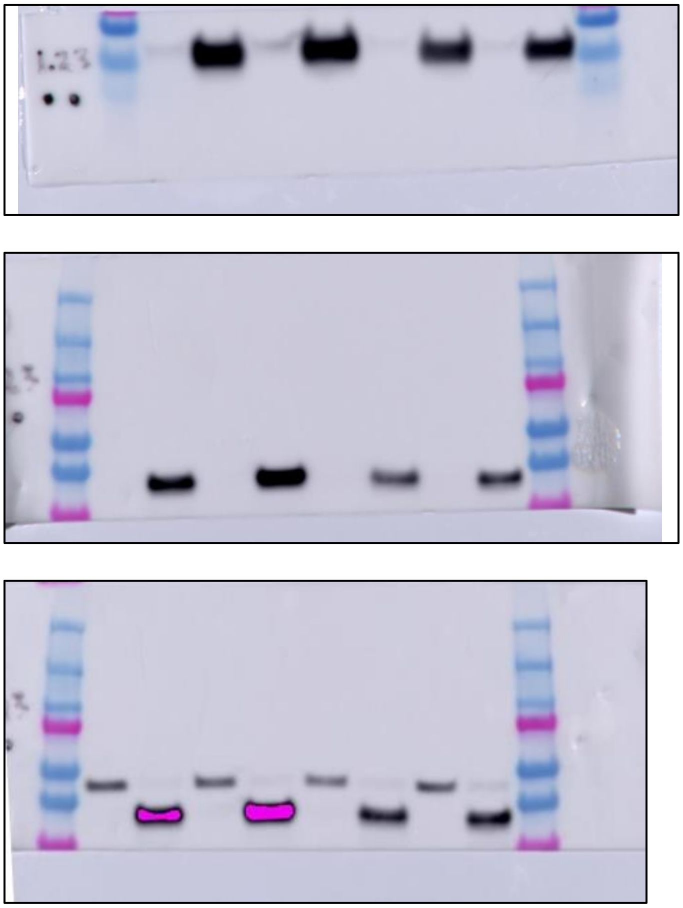

Supplement: Supplementary file 1 — Supplementary Information [file 41467_2022_29039_MOESM1_ESM.pdf]
