# Supplementary material for: Phase II trial of cytarabine and mitoxantrone with devimistat in acute myeloid leukemia
Source: Nat Commun. 2022 Mar 30;13:1673. doi: 10.1038/s41467-022-29039-4 (PMC8967916; doi:10.1038/s41467-022-29039-4)
Supplement: Supplementary file 2 — Reporting Summary [file 41467_2022_29039_MOESM2_ESM.pdf]

nature portfolio

Corresponding author(s): Timothy S Pardee

Last updated by author(s): Jan 12, 2022

Reporting Summary

Nature Portfolio wishes to improve the reproducibility of the work that we publish. This form provides structure for consistency and transparency in reporting. For further information on Nature Portfolio policies, see our Editorial Policies and the Editorial Policy Checklist.

Statistics

For all statistical analyses, confirm that the following items are present in the figure legend, table legend, main text, or Methods section.

n/a	Confirmed	
☐	☒	The exact sample size (*n*) for each experimental group/condition, given as a discrete number and unit of measurement
☐	☒	A statement on whether measurements were taken from distinct samples or whether the same sample was measured repeatedly
☐	☒	The statistical test(s) used AND whether they are one- or two-sided<br>*Only common tests should be described solely by name; describe more complex techniques in the Methods section.*
☐	☒	A description of all covariates tested
☐	☒	A description of any assumptions or corrections, such as tests of normality and adjustment for multiple comparisons
☐	☒	A full description of the statistical parameters including central tendency (e.g. means) or other basic estimates (e.g. regression coefficient) AND variation (e.g. standard deviation) or associated estimates of uncertainty (e.g. confidence intervals)
☐	☒	For null hypothesis testing, the test statistic (e.g. *F*, *t*, *r*) with confidence intervals, effect sizes, degrees of freedom and *P* value noted<br>*Give P values as exact values whenever suitable.*
☒	☐	For Bayesian analysis, information on the choice of priors and Markov chain Monte Carlo settings
☒	☐	For hierarchical and complex designs, identification of the appropriate level for tests and full reporting of outcomes
☐	☒	Estimates of effect sizes (e.g. Cohen's *d*, Pearson's *r*), indicating how they were calculated

Our web collection on statistics for biologists contains articles on many of the points above.

Software and code

Policy information about availability of computer code

Data collection	For RNA sequencing data Adapter contamination was cleaned with Trimmomatic V0.32. Reads were aligned to the reference human genome GRCh38 using the STAR sequence aligner and gene counts determined using featureCounts software version 2.0.3.
Data analysis	Differentially expressed genes were identified by negative binomial modeling using DESeq2 version 3.14 and false discovery correction (q<0.05, Benjamini-Hochberg) Genes associated with patient age (by Pearson correlation) were identified by Gene Set Enrichment Analysis (GSEA). Flow cytometry data was analyzed using FCS Express software, version 7.12.0005. All statistical analysis was done using Graph Pad Prism version 8.3.0 (Graph Pad Software Inc). Combinatorial indices were calculated using Calcusyn version 2.0.

For manuscripts utilizing custom algorithms or software that are central to the research but not yet described in published literature, software must be made available to editors and reviewers. We strongly encourage code deposition in a community repository (e.g. GitHub). See the Nature Portfolio guidelines for submitting code & software for further information.

Data

Policy information about availability of data

All manuscripts must include a data availability statement. This statement should provide the following information, where applicable:

- Accession codes, unique identifiers, or web links for publicly available datasets
- A description of any restrictions on data availability
- For clinical datasets or third party data, please ensure that the statement adheres to our policy

The reference human genome GRCh38 can be found at https://www.ncbi.nlm.nih.gov/assembly/GCF_000001405.39. RNA seq data will be deposited in the Gene Expression Omnibus (GEO). Source data are provided with this paper.

Field-specific reporting

Please select the one below that is the best fit for your research. If you are not sure, read the appropriate sections before making your selection.

☒ Life sciences ☐ Behavioural & social sciences ☐ Ecological, evolutionary & environmental sciences

For a reference copy of the document with all sections, see nature.com/documents/nr-reporting-summary-flat.pdf

Life sciences study design

All studies must disclose on these points even when the disclosure is negative.

Sample size

The primary objective of determining the feasibility of administering CPI-613 in combination with high dose cytarabine and mitoxantrone during induction, consolidation and maintenance therapies will be completed by determining the percentage of patients eligible for maintenance therapy who complete at least 3 cycles. If ≥50% of eligible patients complete 3 cycles of maintenance therapy we will consider this regimen feasible for future study. Given an estimated response rate of 50% and a 25% attrition to stem cell transplant, if we enroll 60 patients we should be able to assess feasibility of maintenance in approximately 22 patients. With 22 evaluable patients, a two-sided 95.0% confidence interval using the large sample normal approximation will extend 22% in either direction from the observed value when the expected value is 50%. Within a dose we should be able to estimate feasibility in 7 patients with a 95% confidence interval extending 36% in either direction.

For mouse experiments sample size was based on previous experience with the syngeneic model to give a 90% power to detect a 1 day difference in median survival. For patient PDX experiments sample size was based on availability of patient derived sample cell numbers.

Data exclusions

No data was excluded.

Replication

All experiments were done in at least 3 separate experiments except for those involving patient samples harvested from mice where separate experiments were not feasible. All experiments that did not have biological replicates had technical replicates. All technical replicates where data was interpretable were successful.

Randomization

Animals were randomly assigned a treatment group following injection of leukemia. Randomization was not applicable to cell culture experiments.

Blinding

Blinding was not applicable to the single arm phase II clinical study. For the mouse experiments blinding was not feasible with the staff and resources available. Blinding is not applicable to cell culture experiments.

Reporting for specific materials, systems and methods

We require information from authors about some types of materials, experimental systems and methods used in many studies. Here, indicate whether each material, system or method listed is relevant to your study. If you are not sure if a list item applies to your research, read the appropriate section before selecting a response.

Materials & experimental systems

n/a	Involved in the study	
☐	☒	Antibodies
☐	☒	Eukaryotic cell lines
☒	☐	Palaeontology and archaeology
☐	☒	Animals and other organisms
☐	☒	Human research participants
☐	☒	Clinical data
☒	☐	Dual use research of concern

Methods

n/a	Involved in the study	
☒	☐	ChIP-seq
☐	☒	Flow cytometry
☒	☐	MRI-based neuroimaging

Antibodies

Antibodies used

Antibodies against TOM20 (Cell Signaling, #42406; 1:1000), VDAC (Abeam, ab14734; 1:1000), and β actin (Abeam, ab8227; 1:2000) were used.

Validation

All antibodies were validated for use in western blotting in mouse and human samples by the manufacturer.

Eukaryotic cell lines

Policy information about cell lines

Cell line source(s)	RHRAS cells were a kindly provided by Dr Gang Greg Wang, RN2 cells by Dr Christopher R. Vakoc. K562 and OCI-AML3 cells were obtained from ATCC.
Authentication	STR analysis was determined every 6 months.
Mycoplasma contamination	All cell lines were tested for mycoplasma every 3 months while in culture and all tested negative.
Commonly misidentified lines (See ICLAC register)	No commonly misidentified cell lines were used in this study.

Animals and other organisms

Policy information about studies involving animals; ARRIVE guidelines recommended for reporting animal research

Laboratory animals	Female C57Bl/6 mice (Jackson Laboratories) 8 to 10 weeks of age were used in this study. Female NSGS mice (Jackson Laboratories) 8 week old were used in this study.
Wild animals	No wild animals were used.
Field-collected samples	No field collected samples were used.
Ethics oversight	The study was approved by the Atrium Health Wake Forest Baptist Animal Care and Use Committee.

Note that full information on the approval of the study protocol must also be provided in the manuscript.

Human research participants

Policy information about studies involving human research participants

Population characteristics	See table 1 for population demographics.
Recruitment	Patients with relapsed or refractory AML seen at the Comprehensive Cancer Center of Atrium Health Wake Forest Baptist were recruited to participate in the study. This population is reflective of the primary Caucasian population in our catchment area and may not reflect the outcomes in minority populations.
Ethics oversight	The study was approved by the Institutional Review Board of the Comprehensive Cancer Center of Atrium Health Wake Forest Baptist and conducted under the supervision of the Safety and Toxicity Review Committee of the Comprehensive Cancer Center of Atrium Health Wake Forest Baptist.

Note that full information on the approval of the study protocol must also be provided in the manuscript.

Clinical data

Policy information about clinical studies

All manuscripts should comply with the ICMJE guidelines for publication of clinical research and a completed CONSORT checklist must be included with all submissions.

Clinical trial registration	NCT02484391
Study protocol	Complete study protocol is provided in the supplementary materials.
Data collection	All data was collected during clinical encounters at the Comprehensive Cancer Center of Atrium Health Wake Forest Baptist. The first patient was enrolled on 10/27/2015 and the last patient was enrolled on 9/28/2018.
Outcomes	Primary Objective: To determine the feasibility of CPI-613 when administered with high dose cytarabine, and mitoxantrone in all three phases of salvage therapy (induction, and maintenance). The regimen will be considered feasible if ≥ 50% of patients eligible for maintenance therapy complete at least 3 cycles.<br>Secondary Objectives: To observe the response rate (CR, and CRi) of CPI-613 in combination with high dose cytarabine and mitoxantrone. This was assessed by bone marrow biopsies according to the by standard criteria for AML (Blood. 2010;115:453-474). To observe the overall survival of patients treated with CPI-613 in combination with high dose cytarabine and mitoxantrone in induction, consolidation and maintenance. This was assessed by clinical visits and follow up phone calls when possible. To monitor toxicities experienced by patients treated with CPI-613 in combination with high dose cytarabine and mitoxantrone in induction, consolidation and maintenance. This was assessed by clinical and laboratory assessments of patients as outlined in the protocol (see supplemental materials).

Flow Cytometry

Plots

Confirm that:

☒ The axis labels state the marker and fluorochrome used (e.g. CD4-FITC).

☒ The axis scales are clearly visible. Include numbers along axes only for bottom left plot of group (a 'group' is an analysis of identical markers).

☒ All plots are contour plots with outliers or pseudocolor plots.

☒ A numerical value for number of cells or percentage (with statistics) is provided.

Methodology

Sample preparation	Patient derived AML cells were purified using CD34 magnetic beads (Miltenyi Biotec) and amplified by passage through NOD.SCIDNSGS mice (Jackson Laboratories). CD34+ cells were injected into sub-lethally irradiated (2 Gy) mice. Peripheral blood was monitored longitudinally for human CD33+ cells. Once substantial engraftment was recorded in the mice the spleens and bone marrow was harvested and the mouse cells were depleted by staining with mouse CD45.l and selected with APC beads (Miltenyi Biotec), and assessed the selection by flow cytometry.
Instrument	Acuri Flow Cytometer
Software	FCS Express software, version 7.12.0005
Cell population abundance	No cell sorting was completed.
Gating strategy	Viable cells were gated by forward scatter versus side scatter.

☒ Tick this box to confirm that a figure exemplifying the gating strategy is provided in the Supplementary Information.
